# Outer Membrane Proteome of *Veillonella parvula:* A Diderm Firmicute of the Human Microbiome

**DOI:** 10.3389/fmicb.2017.01215

**Published:** 2017-06-30

**Authors:** Daniel I. Poppleton, Magalie Duchateau, Véronique Hourdel, Mariette Matondo, Jennifer Flechsler, Andreas Klingl, Christophe Beloin, Simonetta Gribaldo

**Affiliations:** ^1^Unité de Biologie Moléculaire du Gène chez les Extrêmophiles, Département de Microbiologie, Institut PasteurParis, France; ^2^Unité de Spectrométrie de Masse Structurale et Protéomique, Plateforme Protéomique, Départment de Biologie Structurale et Chime, Institut Pasteur, USR 2000 Centre National de la Recherche ScientifiqueParis, France; ^3^Pflanzliche Entwicklungsbiologie und Elektronenmikroskopie, Department I. Botanik, Biozentrum der LMU MünchenPlanegg-Martinsried, Germany; ^4^Unité de Génétique des Biofilms, Département de Microbiologie, Institut PasteurParis, France

**Keywords:** Negativicutes, proteomics, BAM/TAM complex, adhesins, OmpM, LPS, Lpt system, O-antigen

## Abstract

*Veillonella parvula* is a biofilm-forming commensal found in the lungs, vagina, mouth, and gastro-intestinal tract of humans, yet it may develop into an opportunistic pathogen. Furthermore, the presence of *Veillonella* has been associated with the development of a healthy immune system in infants. *Veillonella* belongs to the Negativicutes, a diverse clade of bacteria that represent an evolutionary enigma: they phylogenetically belong to Gram-positive (monoderm) Firmicutes yet maintain an outer membrane (OM) with lipopolysaccharide similar to classic Gram-negative (diderm) bacteria. The OMs of Negativicutes have unique characteristics including the replacement of Braun's lipoprotein by OmpM for tethering the OM to the peptidoglycan. Through phylogenomic analysis, we have recently provided bioinformatic annotation of the Negativicutes diderm cell envelope. We showed that it is a unique type of envelope that was present in the ancestor of present-day Firmicutes and lost multiple times independently in this phylum, giving rise to the monoderm architecture; however, little experimental data is presently available for any Negativicutes cell envelope. Here, we performed the first experimental proteomic characterization of the cell envelope of a diderm Firmicute, producing an OM proteome of *V. parvula*. We initially conducted a thorough bioinformatics analysis of all 1,844 predicted proteins from *V. parvula* DSM 2008's genome using 12 different localization prediction programs. These results were complemented by protein extraction with surface exposed (SE) protein tags and by subcellular fractionation, both of which were analyzed by liquid chromatography tandem mass spectrometry. The merging of proteomics and bioinformatics results allowed identification of 78 OM proteins. These include a number of receptors for TonB-dependent transport, the main component of the BAM system for OM protein biogenesis (BamA), the Lpt system component LptD, which is responsible for insertion of LPS into the OM, and several copies of the major OmpM protein. The annotation of *V. parvula's* OM proteome markedly extends previous inferences on the nature of the cell envelope of Negativicutes, including the experimental evidence of a BAM/TAM system for OM protein biogenesis and of a complete Lpt system for LPS transport to the OM. It also provides important information on the role of OM components in the lifestyle of *Veillonella*, such as a possible gene cluster for O-antigen synthesis and a large number of adhesins. Finally, many OM hypothetical proteins were identified, which are priority targets for further characterization.

## Introduction

*Veillonella parvula*, an anaerobic coccus, was discovered and described 120 years ago when Veillon and Zuber isolated it from an appendicitis abscess (Veillon and Zuber, 1898). Sixty years later the same microbe was used in the first observation of an outer membrane (OM) (Bladen and Mergenhagen, 1964) thereby demonstrating a key difference between the Gram-negative and Gram-positive cell envelope architecture. Since then, 13 other *Veillonella* species have been isolated and described from both humans and rodents (Euzeby, 1997). *Veillonella* strains are found in several niches of the human body including the mouth (Do et al., 2015), lungs, gastrointestinal tract (Rosen et al., 2014), and vagina (Africa et al., 2014). *V. parvula* may serve an important role in many of these environments, however its dominant niche is as a secondary colonizer in the mouth (Griffen et al., 2012). This normal component of the human microbiome may be an opportunistic pathogen; its presence has been associated with several disease states and was the primary infectious agent in at least fifty cases since the 1970's, both with and without an underlying condition (Hirai et al., 2016). In direct opposition to its role as an opportunistic pathogen, recent data suggests that *Veillonella* may perform a protective role and aid in early childhood immune system development. Epidemiological studies of infants have demonstrated that presence of *Veillonella* is negatively correlated with asthma (Arrieta et al., 2015), bronchiolitis (Hasegawa et al., 2016), and autism (Strati et al., 2017).

In addition to being an important component of the human microbiome, *Veillonella*, and other members of the Negativicutes are evolutionary enigmas. They phylogenetically belong to the Firmicutes (low GC Gram-positives or monoderms), yet possess an OM with lipopolysaccharides (LPS) similar to Gram-negative (diderm) bacteria (Zeikus et al., 1983; Tocheva et al., 2011; Campbell et al., 2014). We recently showed that the genomes of Negativicutes contain genes encoding typical diderm OM machinery including flagellar rings typical of diderms, Type 4 pili (T4P) secretin, and an ancestral form of the BAM/TAM system for OM β-barrel assembly (Antunes et al., 2016). Furthermore, most OM-related genes were found to reside in a single genomic cluster (Antunes et al., 2016), hereafter referred to as the “diderm cluster,” that included genes responsible for LPS biosynthesis. Through phylogenomic analysis, we proposed that the diderm cell envelope of Negativicutes represents an ancestral characteristic that was present in the ancestor of all Firmicutes, and was lost multiple times independently to give rise to the monoderm cell envelope in this phylum (Antunes et al., 2016). Although this work presented important perspectives on the OM of *Veillonella*, it was performed *in silico* and cannot tell us which proteins are true components of the OM and whether they are expressed. These predictions can be validated using experimental proteomic approaches, such as those performed on *Bacteroides fragilis* (Wilson et al., 2015) and *Actinobacillus pleuropneumoniae* (Chung et al., 2007).

Only two outer membrane proteins from the Negativicutes have been cloned and studied: OmpM and hemagglutinin-like adhesins. OmpM is an alternative method for tethering the OM to the peptidoglycan by binding of polyamine-modified peptidoglycan through an S-Layer homology (SLH) domain and a transmembrane β-barrel for OM attachment (Kojima and Kamio, 2012). This contrasts with *Escherichia coli's* Braun's lipoprotein (Lpp), which covalently binds PG and integrates into the OM via a lipid moiety (Braun and Rehn, 1969). In the case of adhesins, eight were found in *Veillonella atypica*; one of which, Hag1, was shown to bind human buccal cells and oral streptococci (Zhou et al., 2015).

In order to extend these bioinformatic and experimental data, we have performed the first proteomic analysis of the OM of *V. parvula*. By combining bioinformatic prediction and subcellular fractionation, we have obtained fundamental insight into the nature of these unique cell envelopes and the lifestyle of *Veillonella*, which will help future work on this important component of the human microbiome.

## Materials and methods

### Bioinformatic prediction

To perform *in silico* prediction, we used 12 distinct bioinformatic programs on all 1,844 proteins encoded in the *V. parvula* DSM 2008 genome. Initial prediction was performed using three general prediction programs for IM (Inner Membrane), cytoplasmic, periplasmic, secreted, and OM: PSORTb 3.0.2 (Yu et al., 2010) using default input parameters for Gram-negative bacteria, CELLO 2.5 (Yu et al., 2004) with default input parameters for Gram-negative bacteria, and SOSUI-GramN (Imai et al., 2008) with default parameters. These analyses were complemented with prediction of transmembrane helices by TMHMM 2.0 (Krogh et al., 2001). β-Barrels were predicted using BOMP (Berven et al., 2004) with the additional BLAST option. LipoP 1.0 (Juncker et al., 2003) was used to refine cytoplasmic and IM prediction. Positive lipoprotein prediction was defined as a consensus from PRED-LIPO (Bagos et al., 2008) and LipoP. TAT secreted proteins were identified as a consensus from PRED-TAT (Bagos et al., 2010) and TatP (Bendtsen et al., 2005). Positive SEC signal sequences were defined as a two out of three or greater concurrent result of SignalP, PRED-LIPO, and Phobius (Kall et al., 2007). Extended signal peptides (ESP) were queried using HMMER (Mistry et al., 2013) with the ESPR domain (PF13018) from PFAM within an E-value cutoff of 0.001.

Positive prediction for cytoplasmic proteins was determined by a three out of four or greater concurrent results of PSORT, CELLO, LipoP, and SOSUI. IM prediction was determined by three out of five or greater concurrent results of PSORT, CELLO, TMHMM, LipoP, and SOSUI. Positive OM prediction was determined by three out of four or greater concurrent results of PSORT, CELLO, BOMP, and SOSUI. Positive periplasmic prediction and secreted proteins were determined by a two out of three or greater concurrent results of PSORT, CELLO, and SOSUI.

### Search for conserved domains and homologs

To identify homologs of experimentally identified proteins we ran a BLAST 2.2.30+ (Camacho et al., 2009) search against two local databases. The first contains a selection of 255 representative Firmicutes and the second contains all 112 currently available Negativicutes genomes. Default settings were used except for an E-value cutoff of 0.0001. Protein domains were searched using the conserved domain database (CDD) from NCBI (Marchler-Bauer et al., 2014), PFAM (Finn et al., 2015), InterPro (Finn et al., 2017), and PANTHER 10.0 (Mi et al., 2015) with the required E-value cutoff of 0.00001. Protein folds were predicted with Phyre2 (Kelley et al., 2015).

### Outer membrane protein extraction

The extraction protocol was modified from Thein et al. (2010). Fifty milliliters of *V. parvula* DSM 2008 were grown anaerobically in triplicate to an optical density at 600 nm of 0.4 (10 h after a 1:100 dilution) in BHIL (BHI (Bacto) + 0.6% sodium L-lactate). The bacteria were harvested by centrifugation at 15,000 × g for 20 min at 4°C and resuspended in an equal volume of Tris-HCl (0.1 M pH 7.3 supplemented with 7 mg of DNAse). Cells were washed two additional times and suspended in 6 ml of the same buffer. Cells were lysed by French press at 10,000 kPa for four passes in Tris-HCl (20 mM pH 7.3). Cellular debris were pelleted by centrifugation at 15,000 × g for 20 min at 4°C. The supernatant was collected and the pellet was discarded. This step was repeated an additional time. Supernatant was then diluted with ice cold 0.1 M Na_2_CO_3_ pH 11 to a volume of 60 ml and stirred for 1 h at 4°C. The suspension was separated at 120,000 × g for 1 h at 4°C and the pellet washed in an equal volume of Tris-HCl (0.1 M pH 7.3) and spun at 85,000 × g for 20 min at 4°C. The wash was repeated twice. The pellet was resuspended in ddH_2_O and proteins were TCA precipitated before MS analysis.

### Surface exposed and control sample extraction

For extraction of surface exposed (SE) fraction, as well as for control samples, a protocol was modified from Voss et al. (2014). Bacteria were grown in triplicate and harvested as in the OM protein extraction. Five milliliters of bacterial cells were washed three times in PBS (0.1 M PO_4_, 0.15 M NaCl pH 8.0). Cells were resuspended in PBS containing 20 mM of NHS-PEG4-Biotin (Thermo Fisher Scientific) and incubated on ice for 30 min. Reaction was quenched by washing cells in quench buffer (PBS + 100 mM Glycine) three times. Control samples (WC) were treated identically except that they were incubated in PBS without labeling substrate. Cells were lysed by French press at 10,000 kPa for four passes in radioimmunoprecipitation (RIPA) buffer [25 mM Tris-HCl, pH 7.6, 150 mM NaCl, 1% NP-40, 1% sodium deoxycholate, and 0.1% sodium dodecyl sulfate (SDS) containing a 1:100 dilution of protease inhibitors] and cellular debris were removed by centrifugation at 15,000 × g for 20 min at 4°C twice. Control sample was then TCA precipitated. For SE fraction 1 ml high Capacity Streptavidin Resin columns (Thermo Fisher Scientific) and reagents were equilibrated to room temperature. PBS and elution buffer (8 M guanidine-HCl, pH 1.5) were filtered and degassed. Column was equilibrated with five column volumes of PBS at a flow rate of 0.2 mL/min on an AKTA-FPLC. Sample was then applied to the column using the same rate and washed with 10 column volumes of PBS before elution in a single column volume of elution buffer. Eluate then underwent MS analysis.

### Trichloroacetic acid (TCA) precipitation

TCA was added to OM samples and WC samples to a final concentration of 20%. The precipitate was spun at 6,500 × g for 1 h at 4°C. The pellet was washed with 800 μl of −20°C acetone overnight and then spun at 6,500 g for 1 h at 4°C. The washing procedure was performed two additional times before resuspension or storage at −20°C.

### LPS extraction and visualization

*E. coli* strains were kindly provided by Laurent Debarbieux. *E. coli* (81009, 81009Δ*waaF*, E47a, E47aΔ*rfb3*; Szijarto et al., 2014) and *V. parvula* DSM 2008 were grown in triplicate to an OD of 0.4. LPS was isolated using hot phenol extraction without modification as previously described (Davis et al., 2012). LPS was then resolved onto either a 0.1% SDS 13% or 17% PAGE gel and visualized with Pro-Q Emerald 300 (ThermoFisher Scientific) as per the manufacturer's instructions.

### In-gel protein digestion

Protein samples were loaded on a 0.1% SDS 12% PAGE gel. After the electrophoretic migration, the gel was stained with Coomassie Blue, each band of interest was cut, and in-gel tryptic digestion was performed as described previously (Wilm et al., 1996). Briefly, gel slices were washed in 100 mM ammonium bicarbonate for 15 min, followed by several washing steps to eliminate the stain in 100 mM ammonium bicarbonate/acetonitrile (1:1). Samples were reduced (10 mM DTT in 100 mM ammonium bicarbonate, 30 min at 56°C) and alkylated (55 mM iodoacetamide in 100 mM ammonium bicarbonate, 30 min at room temperature in the dark). Proteins were digested by 250 ng Sequencing Grade Modified Trypsin (Promega, Madison, WI, USA) in 10 mM ammonium bicarbonate overnight at 37°C. Resulting peptides were extracted, dried in the Speed-Vac and then resuspended in water/acetronitrile/formic acid (98:2:0.1).

### Liquid protein digestion

Tryptic digestion was performed by eFASP (enhanced Filter-Aided Sample Preparation) as described previously (Erde et al., 2014). Samples were buffer-exchanged into buffer containing 8 M urea, 100 mM ammonium bicarbonate, and 0.2% deoxycholic acid using Tween passivated 30-kDa cut-off Amicon filters (Millipore Inc., USA). Samples were reduced with 5 mM tris (2-carboxyethyl) phosphine (TCEP) for 30 min at room temperature, alkylated in iodoacetamide (50 mM final, 30 min room temperature in the dark), treated with 1 μg of sequencing-grade modified trypsin (Promega, USA) overnight at 37°C under agitation. Peptides were recovered by centrifugation.

### Mass spectrometry analysis

Trypsin-digested peptides coming from gel samples were analyzed by nano LC-MS/MS using an Ultimate 3000 system (Dionex, Amsterdam, The Netherlands) coupled to an LTQ-Orbitrap Velos. File microliters of each sample were loaded on a C_18_ pre-column (300 μm inner diameter × 5 mm; Dionex) at 30 μL/min in 2% ACN, 0.1% FA. After 5 min of desalting, the pre-column was switched online an in-house packed 15 cm nano-HPLC column (75 μm inner diameter) with C_18_ resin (3 μm particles, 100 Å pore size, ReproSil-Pur Basic C_18_, Dr. Maisch GmbH, Ammerbuch-Entringen, Germany) and equilibrated in 95% solvent A (2% ACN, 0.1% FA) and 5% solvent B (80% ACN, 0.08% FA). The peptides were eluted using a 2–50% gradient of solvent B during 60 min at 300 nL/min flow rate. The LTQ-Orbitrap Velos (Thermo Fisher Scientific, Bremen) was operated in data-dependent acquisition mode with the XCalibur software (Thermo Fisher Scientific, Bremen). Survey scan MS were acquired in the Orbitrap on the 300–2,000 m/z range with the resolution set to a value of 60,000 at m/z = 400. The 10 most intense ions per survey scan were selected for CID fragmentation, and the resulting fragments were analyzed in the linear trap (LTQ). The dynamic exclusion was enabled with the following settings: repeat count, 1; repeat duration, 30 s; exclusion list size, 500; and exclusion duration, 20 s.

Digests coming from liquid samples were analyzed on an Orbitrap Q Exactive Plus mass spectrometer (Thermo Fisher Scientific, Bremen) coupled with an EASY nLC 1000 chromatography system (Thermo Fisher Scientific). Sample was loaded on an in-house packed 40 cm nano-HPLC column (75 μm inner diameter) with C_18_ resin (1.9 μm particles, 100 Å pore size, Reprosil-Pur Basic C_18_-HD resin, Dr. Maisch GmbH, Ammerbuch-Entringen, Germany) and equilibrated in 98% solvent A (H_2_O, 0.1% FA) and 2% solvent B (ACN, 0.1% FA). Peptides were eluted using a 2–45% gradient of solvent B during 120 or 240 min at 250 nL/min flow rate. The instrument method for the Q Exactive Plus was set up in the data dependent acquisition mode using XCalibur 2.2 software (Thermo Fisher Scientific, Bremen). After a survey scan in the Orbitrap (resolution 70,000 at m/z 400), the 10 most intense precursor ions were selected for higher-energy collision dissociation (HCD) fragmentation with a normalized collision energy set to 28. Charge state screening was enabled, and precursors with unknown charge state or a charge state of 1 and >7 were excluded. Dynamic exclusion was enabled for 35 s.

In order to increase throughput and sensitivity methods used to acquire the data with these two mass spectrometer have been optimized in accordance with recommended settings from Thermo Fisher Scientific using HeLa extract sample (from Thermo) and an in-house complex quality control sample.

### Data processing and analysis

Raw data were searched using MaxQuant (version 1.4.1.2) (Cox and Mann, 2008; Cox et al., 2014; with the Andromeda search engine) against *V. parvula* Uniprot database (1,844 entries, version 10-2015) and concatenated with known MS contaminants and reversed sequences of all entries. Andromeda searches were performed choosing trypsin as specific enzyme with a maximum number of two missed cleavages. Possible modifications included carbamidomethylation (Cys, fixed), oxidation (Met, variable), and Nter acetylation (variable). Maximum peptide charge was set to seven and five amino acids were required as minimum peptide length. Most peptides (~89%) were identified with charge states < +3. Less than 10% of peptides were identified with a charge state > +4. As you may see on the graph, peptides (~0.1%) were identified with a charge state of +6 (for details see PSM charges Pourcentage file deposited in PRIDE). Andromeda default settings were used; an initial search was performed using a mass tolerance of 20 ppm, followed by mass recalibration and a main search with a mass tolerance of 5 ppm for parent ions (Tyanova et al., 2016a). In the MS/MS search, mass tolerance was set to 10 ppm for the Q Exactive Plus and to 0.5 Da for the LTQ-Orbitrap Velos. Additional peptides were identified by the “match between run” option with a maximal retention time window of 1 min. One unique peptide to the protein group was required for the protein identification. Only unique peptides were used to distinguish isoforms. If no unique peptide was identified, isoforms were combined into the same “protein group” and the quantification was done for this protein group. A false discovery rate (FDR) cutoff of 1% was applied at the peptide and protein levels. MaxLFQ, Maxquant's label-free quantification (LFQ) algorithm was used to calculate protein intensity profiles across samples (Cox et al., 2014). Data were filtered by requiring a minimum peptide ratio count of two in MaxLFQ. Absolute protein amounts were calculated in Maxquant as the sum of all peptide peak intensities divided by the number of theoretically observable tryptic peptides (intensity-based absolute quantification or iBAQ (Schwanhäusser et al., 2011).

For statistical and bioinformatics analysis, as well as for visualization, Perseus environment was used, which is part of Maxquant (Tyanova et al., 2016b).

The “proteinGroup.txt” file generated with Maxquant was used by Perseus to identify proteins enriched in Biotin samples. Protein identifications were filtered, removing hits to the reverse decoy database as well as proteins only identified by modified peptides or considered as potential contaminant. Protein LFQ intensities were logarithmized. Two valid values out of three were required for each protein for the confident quantification across all replicates and missing values imputed by values simulating low abundance values close to the noise level. For pairwise comparison and identification of enriched proteins, a modified *t*-test were applied with permutation-based FDR statistics set to 1% and a S_0_ of 1 (Tusher et al., 2001).

The mass spectrometry proteomics data have been deposited to the ProteomeXchange Consortium via the PRIDE (Vizcaíno et al., 2016) partner repository with the dataset identifier PXD005929.

### Transmission electron microscopy

A densely grown culture of *V. parvula* DSM 2008 was used for the preparation for transmission electron microscopy (TEM). After concentration of the cells via centrifugation, the cells were either chemically fixed with glutaraldehyde or cryo-fixed with a high-pressure freezer and subsequent freeze substitution.

For chemical fixation, the cell pellet was resuspended in a fixation buffer consisting of 100 mM cacodylate including 2 mM MgCl_2_, 2.5% glutaraldehyde (final concentration; 25% stock solution), and 270 mM NaCl to adjust the osmolarity of the buffer to the original growth medium. After fixation, the cells were washed five times with buffer, post-fixed for 30 min in 1% osmium tetroxide, washed again two times with buffer and three times with double distilled water. Afterwards, the cells were dehydrated in a graded acetone series, infiltrated with Spurr‘s resin and polymerized for 72 h at 63°C. The embedded samples were then ultrathin sectioned (50 nm sections), post-stained with lead citrate and visualized in a TEM.

High-pressure freezing was performed with 2 μl of the resuspended concentrated cells in the respective aluminum platelets using a Leica HPM 100 high-pressure freezer (Leica Microsystems GmbH, Wetzlar, Germany). Freeze substitution was performed in a Leica AFS 2 (Leica Microsystems GmbH, Wetzlar, Germany) according to the following protocol (substitution solution): −90°C for 20 h (2% OsO4/acetone), heating to −60°C within 3 h (2% OsO4/acetone), −60°C for 4 h (2% OsO4/acetone), −60°C for 4 h (2% OsO4/0. 2% uranyl acetate/acetone), heating to −30°C within 3 h (2% OsO4/0.2% uranyl acetate/acetone), −30°C for 8 h (2% OsO4/0.2% uranyl acetate/acetone), heating to 0°C (2% OsO4/0.2% uranyl acetate/acetone). After washing three times with ice cold acetone, the samples were infiltrated with epoxy resin (Spurr‘s resin) and polymerized for 72 h at 63°C. The following steps were identical to the chemical fixation protocol.

Transmission electron microscopy was performed on a Zeiss EM 912 (Carl Zeiss AG, Oberkochen, Germany), operated at 80 kV in the zero-loss mode and equipped with a 2 k × 2 k dual-speed CCD camera (Tröndle Restlichtverstärkersysteme, Moorenweis, Germany).

## Results and discussion

### Bioinformatic localization prediction

**Figure 1:**
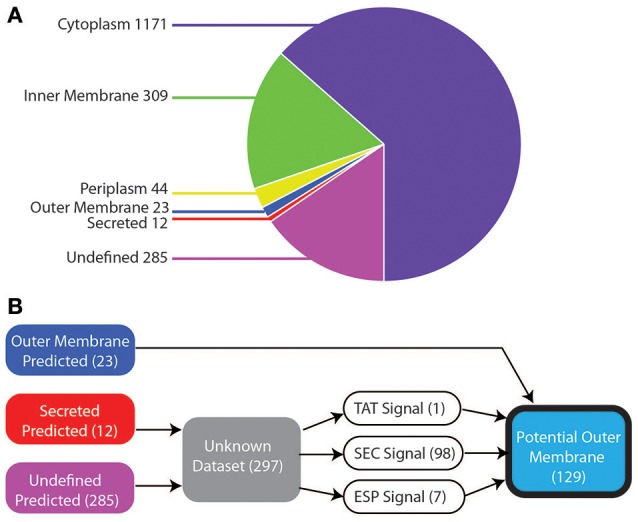
Bioinformatic localization prediction. **(A)** Localization of *V. parvula* proteome predicted by PSORT, CELLO, SOSUI, LipoP, TMHMM, and BOMP (see text for details). **(B)** Strategy for refining prediction for the sequences with undefined localization (see text for details).

We started our study with a comprehensive *in silico* analysis of all 1,844 annotated protein-coding genes within the genome of *V. parvula* DSM 2008 (see Section Materials and Methods). The localization prediction of these 1,844 proteins are shown in Figure 1A. We used three general prediction programs (PSORT, CELLO, and SOSUI) for inner membrane (IM), cytoplasmic, periplasmic, secreted, and OM, and specific programs for OM (BOMP), IM (TMHMM and LipoP), and cytoplasmic (LipoP) localization. With this strategy, we managed to robustly predict the localization of 85% (1559/1844) of the proteins: 63% (1,171) cytoplasmic, 17% (309) IM, 2% (44) periplasmic, 1% (23) OM, 0.5% (12) secreted, whereas 15% (285) proteins remained with undefined localization (Figure 1A and Table S1).

The 23 proteins robustly predicted to be in the OM include many known OM components such as TonB-dependent receptors, OmpM, components of the BAM/TAM machinery, TolC, and OmpA (Table S1). Although localization prediction methods were able to identify these OM components, they have limitations due to training on datasets of known OM proteins, most of which are from the phylogenetically distant Proteobacteria. This could prevent proper prediction of many proteins and fail to find novel OM proteins. Indeed, they failed to correctly identify the experimentally characterized trimeric autotransporters, which were predicted as secreted or had undefined localization (Table S1).

As such, we more thoroughly investigated the 297 proteins with undefined prediction or predicted as secreted, as they might contain additional OM proteins. As shown in Figure 1B, to do so, we constructed a bioinformatic filter to sort these 297 proteins. Briefly, we identified the proteins that contained a signal sequence for translocating through the IM, by checking for the three IM transport mechanisms: SEC, ESP (Extended signal peptide), and TAT with seven different programs (Figure 1B; see Section Materials and Methods). Hundred and ninety-one proteins lacked any recognizable signal sequence and were excluded. The remaining 106, which included all of the previously missed trimeric autotransporters, were combined with the 23 predicted OM proteins to provide a final dataset of 129 potential outer membrane proteins (Table S2).

### Subcellular fractionation

To validate our *in silico* prediction and to firmly identify OM proteins, we carried out a proteomic analysis of the OM of *V. parvula* DSM 2008 (see Section Materials and Methods for details). We performed three extractions: an outer membrane (OM) extract, a surface exposed (SE) extract, and a control whole cell (WC) protein extract. For all three extractions, *V. parvula* was grown anaerobically in BHIL medium in triplicate and the extraction was performed during the exponential growth phase. For OM protein extraction, we performed French press lysis to produce vesicles and isolated the OM vesicles with a chaotropic agent. To identify SE proteins we used a cell impermeable substrate, PEG-Biotin, which would label any primary amine exposed to the external environment of the cell. For each of the three extractions, only proteins that were present in all three biological replicates were considered for further analysis.

**Figure 2:**
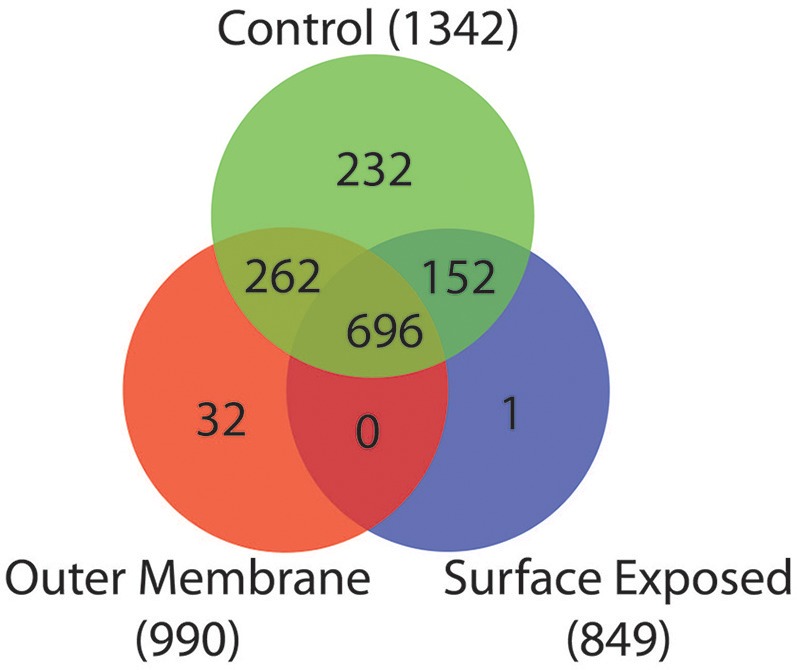
Venn diagram of peptides detected from the three extraction methods. Overlap of peptides detected in the control sample (Green), OM extraction (Red), and SE extraction (Blue).

To identify proteins that were unique or shared by the different fractions we constructed a Venn diagram that is depicted in Figure 2. The WC control extract contained 1,342 proteins, while the OM fraction contained 990 proteins, and the SE fraction contained 849 proteins. Many false positives, including ribosomal proteins, were present in all three fractions (Table S1). This is a known product of the high sensitivity of MS analysis (Pocsfalvi et al., 2016).

Among the 1,342 proteins present in the WC extract, 696 were also detected in both the OM and SE fractions (Figure 2). Two hundred and sixty-two were detected in the WC and OM fractions but not in the SE. These proteins probably lack an exposed primary amine that would make them available to the biotinylation substrate. Similarly, 152 proteins were detected in the WC and SE fractions but not the OM fraction. These included many cytoplasmic or IM components and are therefore possible contaminants (Table S1). Finally, we found 32 proteins unique to the OM extract, and one protein unique to the SE extract. These discrepancies may be due to the enrichment of specific proteins by the OM and SE extraction protocols, combined with a too low concentration for detection in the WC control sample.

**Figure 3:**
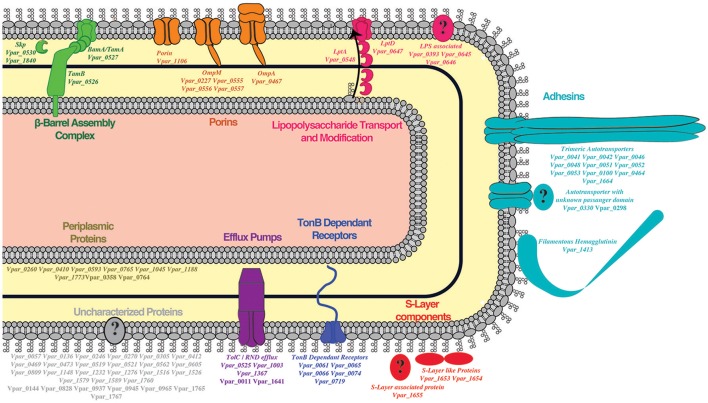
Outer membrane proteome schematic demonstrating the different systems detected by our methods. Proteins found in the OM are in bold while SE detection is indicated by bold and italics.

For the remainder of the analysis we only considered proteins as potential OM if they were detected in both the OM and the WC control samples, while we considered proteins as potential SE if they were present in all three samples, leading to 958 proteins (WC + OM = 262, WC + OM + SE = 696). In order to reduce false positives from this dataset, we crossed it with the 129 potential OM proteins obtained by our bioinformatics prediction (Table S2). This led to a final list of 78 OM proteins (Table 1 and Table S3) that we annotated and created a graphical schematic representation in a *V. parvula* cell as shown in Figure 3. Identification of these OM proteins markedly extends previous inferences on the nature of the cell envelope of Negativicutes (Antunes et al., 2016) and provides information on the role of OM systems in the lifestyle of *Veillonella*.

**Table 1:** 78 OM proteins identified by bioinformatic localization and proteomic analysis. Order follows discussion in text.

**Gene ID**	**Gene description**	**Robust prediction**	**WC**	**OM**	**SE**
Vpar_0526	TamB	OM	+	+	+
Vpar_0527	BamA/TamA	OM	+	+	+
Vpar_0530^*^	Skp (OmpH)	Unclear	+	+	+
Vpar_1840	Skp (OmpH)	Unclear	+	+	+
Vpar_1106	Porin	Unclear	+	+	+
Vpar_0227	OmpM	OM	+	+	+
Vpar_0555	OmpM	OM	+	+	+
Vpar_0556	OmpM	OM	+	+	+
Vpar_0557	OmpM	OM	+	+	+
Vpar_0467	OmpA	OM	+	+	+
Vpar_0647	LptD	OM	+	+	+
Vpar_0548	LptA	Unclear	+	+	+
Vpar_0645	Uncharacterized protein	Unclear	+	+	+
Vpar_0646	Uncharacterized protein	Unclear	+	+	+
Vpar_0393	Uncharacterized protein	OM	+	+	+
Vpar_0051	Trimeric autotransporter adhesin	Secreted	+	+	+
Vpar_0052	Trimeric autotransporter adhesin	Secreted	+	+	+
Vpar_0464	Trimeric autotransporter adhesin	Unclear	+	+	+
Vpar_0048	Trimeric autotransporter adhesin	Unclear	+	+	+
Vpar_0042	Trimeric autotransporter adhesin	Unclear	+	+	+
Vpar_0046	Trimeric autotransporter adhesin	Unclear	+	+	+
Vpar_1413	TpsA	Unclear	+	+	+
Vpar_0041	Trimeric autotransporter adhesin	Unclear	+	+	+
Vpar_0100	Trimeric autotransporter adhesin	Unclear	+	+	+
Vpar_1664	Trimeric autotransporter adhesin	Unclear	+	+	+
Vpar_0053	Hemagglutinin domain protein	Unclear	+	+	+
Vpar_0330	Autotransporter	OM	+	+	+
Vpar_0298	Autotransporter-ShdA like	Secreted	+	+	
Vpar_1653	S-layer domain protein	Unclear	+	+	+
Vpar_1654	S-layer domain protein	Unclear	+	+	+
Vpar_1655	Uncharacterized protein	Unclear	+	+	+
Vpar_0074	TonB family protein	OM	+	+	+
Vpar_0061	TonB family protein	OM	+	+	+
Vpar_0065	TonB family protein	OM	+	+	+
Vpar_0066	TonB family protein	OM	+	+	+
Vpar_0719	TonB family protein	OM	+	+	+
Vpar_0525	TolC	OM	+	+	+
Vpar_1367	TolC	OM	+	+	+
Vpar_1003^*^	RND family efflux pump	Unclear	+	+	+
Vpar_0011^*^	RND family efflux pump	Unclear	+	+	
Vpar_1641^*^	Efflux transporter, RND family, MFP subunit	Unclear	+	+	
Vpar_0270	Peptidase: PepSY family	Secreted	+	+	+
Vpar_0057	Peptidase M48 family	Unclear	+	+	+
Vpar_0246	Peptidase: PepSY family	Unclear	+	+	+
Vpar_0412	Uncharacterized protein	Unclear	+	+	+
Vpar_0605	Peptidoglycan binding protein	Unclear	+	+	+
Vpar_1232^*^	Uncharacterized protein	Unclear	+	+	+
Vpar_1579	ATPase involved in DNA repair-like protein	Unclear	+	+	+
Vpar_1589^*^	Unknown Protein containing DUF3829 domain	Unclear	+	+	+
Vpar_0136	Unknown protein containing copper amine oxidase domain	Unclear	+	+	+
Vpar_0305	Uncharacterized protein	Unclear	+	+	+
Vpar_0469	Peptidase: PepSY family	Unclear	+	+	+
Vpar_0473	Unknown Protein containing DUF541 domain	Unclear	+	+	+
Vpar_0519	Unknown Protein containing DUF2233 domain	Unclear	+	+	+
Vpar_0521	Unknown protein	Unclear	+	+	+
Vpar_0562	Unknown protein containing DUF1421 domain	Unclear	+	+	+
Vpar_1148	Uncharacterized protein	Unclear	+	+	+
Vpar_1276	Uncharacterized protein	Unclear	+	+	+
Vpar_1760^*^	Unknown protein containing DUF3829 domain	Unclear	+	+	+
Vpar_1516	Uncharacterized protein	Unclear	+	+	+
Vpar_0809	Peptidase M23	Unclear	+	+	+
Vpar_1526	Uncharacterized protein	Unclear	+	+	+
Vpar_1767^*^	TPR repeat-containing protein	Secreted	+	+	
Vpar_0144	Hypothetical: MAEBL like protein	Unclear	+	+	
Vpar_0937^*^	VanW like protein	Unclear	+	+	
Vpar_0945^*^	Uncharacterized protein	Unclear	+	+	
Vpar_0965	Glycoside hydrolase family 18	Unclear	+	+	
Vpar_1765^*^	Unknown protein containing DUF3829 domain	Unclear	+	+	
Vpar_0828	HI0933 family protein	Unclear	+	+	
Vpar_0593	SpoIID/LytB domain protein	Secreted	+	+	+
Vpar_0260^*^	Periplasmic binding protein	Unclear	+	+	+
Vpar_0765^*^	Periplasmic binding protein	Unclear	+	+	+
Vpar_0410^*^	Periplasmic binding protein	Unclear	+	+	+
Vpar_1045^*^	Beta-lactamase domain protein	Unclear	+	+	+
Vpar_1188	Hydrogenase (NiFe) small subunit HydA	Unclear	+	+	+
Vpar_1773	Beta-N-acetylhexosaminidase (EC 3.2.1.52)	Unclear	+	+	+
Vpar_0358^*^	Periplasmic binding protein	Unclear	+	+	
Vpar_0764^*^	Periplasmic binding protein	Unclear	+	+	

* *Predicted lipoproteins that may be anchored to the inner membrane*.

Additionally, among the 1,844 predicted proteins of *V. parvula* roughly one quarter of them (502) were not expressed in our growth conditions or were not detected by MS due to their low concentration. As an example, we previously detected a conserved genomic locus coding for all components of a Type 4 pilus (T4P), including an OM secretin (Antunes et al., 2016). This cluster is similar to characterized Type 4A pili, which are known to be responsible for twitching motility (Pelicic, 2008) and natural competence (Knapp et al., 2017). However, we could only detect three peptides of PilM in one of the three WC control extraction replicates and three peptides of the component PilA in one of the three OM extraction replicates (Table S4). All remaining T4P components were absent in all of our samples, demonstrating the absence of their production in our growth conditions. Nonetheless, a T4P and the other undetected proteins may be produced in other conditions such as *in vivo* or during biofilm formation. Indeed, other species of *V. parvula* have shown differential competence capabilities in different media; this is likely related to T4P expression (Knapp et al., 2017).

### An ancestral β-barrel assembly machinery, BAM/TAM, is present and functional in the OM of *V. parvula*

The detection of OM proteins implies the existence of a functional system to insert them in the OM. The presence of a peculiar and probably ancestral BAM/TAM machinery has been inferred in *Negativicutes* and other bacteria (Webb et al., 2012; Heinz et al., 2015; Antunes et al., 2016). This system putatively contains three components, the BamA/TamA assembly barrel (Omp85), the TAM IM/periplasmic chaperone (TamB), and the BAM periplasmic chaperone (Skp). It has been hypothesized that this ancestral BAM/TAM machinery functions as both BAM and TAM in diderm Firmicutes (Heinz et al., 2015; Antunes et al., 2016). This hypothesis is supported by the detection of only a single Omp85 homolog in each genome and the fact all these genes form a conserved genomic cluster, suggesting functional linkage. Indeed, a recent study on TamB from *Borrelia burgdorferi* demonstrated that it interacts with BamA composing a BAM/TAM system (Iqbal et al., 2016).

Twenty-one of the 23 robustly predicted OM proteins were predicted as β-barrels, of which 17 were detected in our OM. This provides the first experimental evidence that the BAM/TAM system is functional in *V. parvula*. Moreover, we detected the major OM component BamA/TamA (Vpar_0527), as well as TamB (Vpar_0526) in both our SE and OM fractions (Figure 3, Table 1, and Table S3). While the presence of the OM component BamA/TamA was expected, that of TamB is surprising, as TamB in other species is known to be periplasmic and attached to the IM. One can speculate that in *V. parvula* an entire complex containing BamA/TamA and TamB may be captured in the OM fraction. However, this fails to account for the strong OM prediction of TamB by localization software PSORT, CELLO, and SOSUI (Table S1), which indicates that some OM characteristics must be present within the *V. parvula* TamB; unfortunately we cannot determine what these characteristics are, due to the machine learning nature of the programs. Interestingly, TamB of *E. coli* (NP_418642.1) is also predicted to be in the OM by two out of the three programs (not shown), yet its IM/periplasmic localization has been firmly established through protease shaving (Selkrig et al., 2012). Discrepancies also exist in previous fractionation mass-spectrometry studies, as TamB was found in higher concentration in the OM than in the IM in *E. coli* (Martorana et al., 2014). Together, these data suggest that TamB may actually be both in the OM and the IM, or span the two membranes.

Four genes potentially encode the chaperone Skp in the *V. parvula* DSM 2008 genome (*vpar_0528-30, vpar_1840*), three of which are present in the diderm cluster immediately downstream of the BamA/TamA gene (Antunes et al., 2016). All four Skp proteins were detected in our OM fraction. However, two of them were excluded by our localization prediction procedure because of their strong prediction as periplasmic proteins (Table S1). Presence of Skp in the OM fraction is not unusual as Skp in *Salmonella* was originally thought to be in the OM, hence the old name OmpH (Thome and Müller, 1991). Furthermore, it has been shown experimentally that this protein interacts strongly with both the BAM system and OmpA, by functioning as a chaperone helping insertion of proteins in the OM (Selkrig et al., 2014).

### Porins are a fundamental component of *Veillonella*'s outer membrane

A key component of bacterial OM are porins, which are necessary for passive diffusion of small molecules (Galdiero et al., 2012). The genome of *V. parvula* contains genes encoding a typical porin (*vpar*_1106) consisting entirely of a β-barrel, four copies of OmpM (*vpar_0227, vpar_0555-7*) which contains a porin domain and a periplasmic SLH domain, and a homolog of the pathogenicity factor OmpA (*vpar_0467*) containing typical OmpA and beta-barrel domains (Confer and Ayalew, 2013).

We previously described the presence of three OmpM encoding genes in tandem within the diderm cluster of *V. parvula* (*vpar_0555-7*) (Antunes et al., 2016). During the course of this analysis we discovered a fourth (*vpar_0227*) at a different locus. The three OmpM proteins found within the diderm cluster (Vpar_0555-7) are among the most abundant proteins in the OM fraction by both quantification of our LC-MS/MS data (Table S5) and SDS-PAGE band excision MS (Bands 4 and 5) (Table S6). This correlates well with the function as an OM tether, as in *E. coli* Braun's lipoprotein is the most numerous protein in the cell (Braun, 1975). Furthermore, this supports the hypothesis that all Negativicutes utilize OmpM as a form of attachment (Kojima and Kamio, 2012). Moreover, this is consistent with a transcriptomics study of three *Veillonella* species from the mouth, which found all three copies of OmpM to be the most abundant OM transcripts (Do et al., 2015), further highlighting the importance of these proteins (Kojima et al., 2016).

We detected all four copies of OmpM in both the OM and the SE fractions (Figure 3, Table 1, and Table S3). Because of OmpM's abundance and dominant role in the OM, an exposed area of *Veillonella*'s OmpM may be involved in adhesion and biofilm formation. Previous bioinformatics studies have suggested that OmpM in the Negativicute S*elenomonas ruminantium* may be SE and interact with other bacteria (Kojima et al., 2011). We previously wondered why there are multiple copies of the OmpM tether in diderm Firmicutes (Antunes et al., 2016); indeed, Braun's lipoprotein is usually in single copy, with the exception of *Salmonella's* from a recent gene duplication event (Sha et al., 2004). Because the four OmpM proteins are not identical, it is possible that they each provide a different surface for adhesion and/or immune evasion.

**Figure 4:**
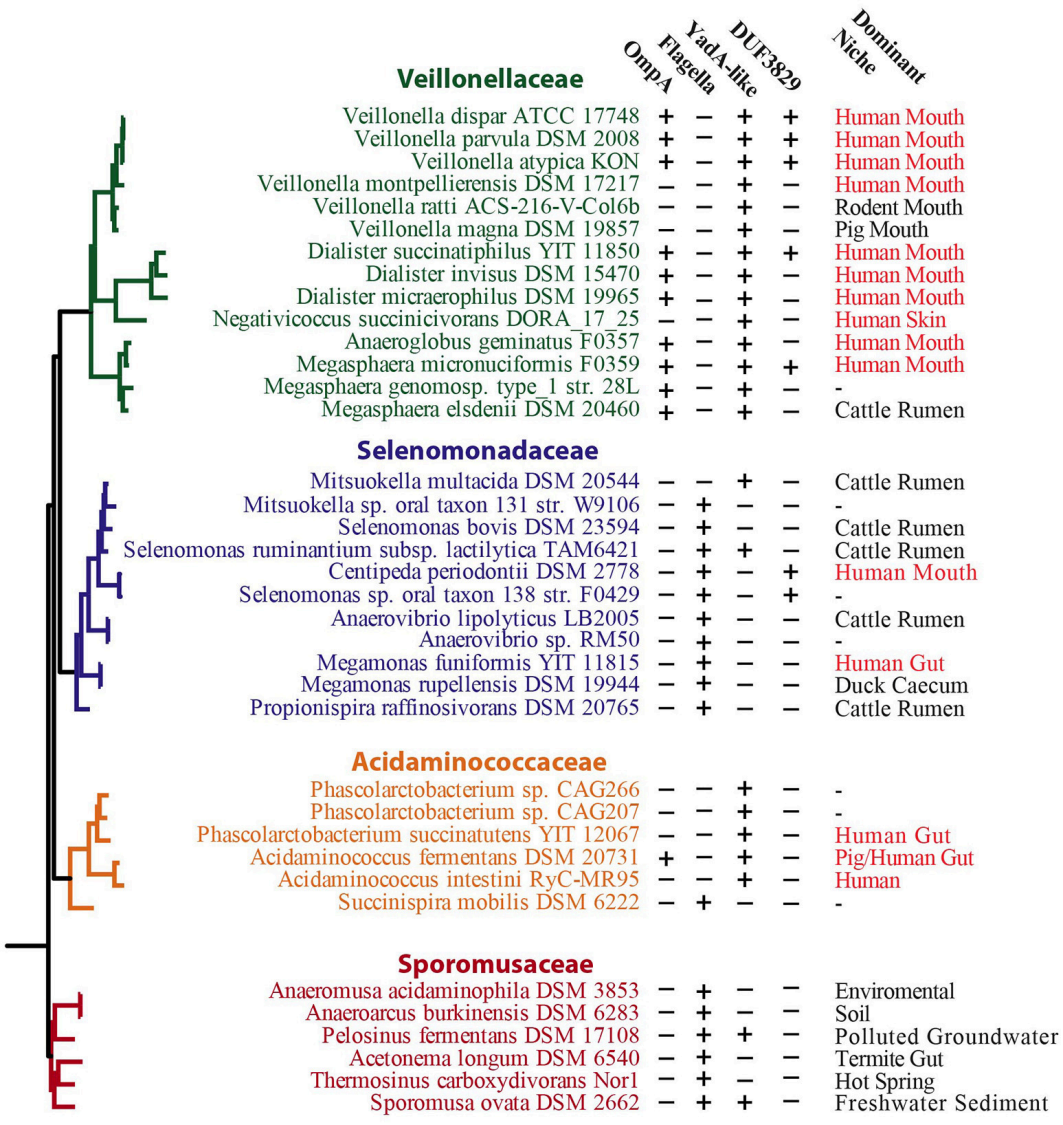
Distribution of selected protein-coding genes in select proteins among Negativicutes. Schematic tree based on phylogeny of the Negativicutes from previous analysis (Antunes et al., 2016). Presence and absence of key genes are indicated by + and − symbols, respectively. The dominant niche of each strain taken from the available literature is also given. For discussion, see main text.

We also detected an OmpA homolog (Vpar_0467) in the OM and SE fractions. OmpA is an important protein in the OM of bacteria as it has strong pathogenic roles involving cellular invasion, adhesion, and host cell evasion (Confer and Ayalew, 2013) and therefore may be involved specifically in pathogenicity of *V. parvula*. Consistent with this hypothesis, a search for OmpA homologs in other Negativicutes revealed its presence in a few other human-related members of the family Veillonellaceae (Figure 4).

### A complete LPS transport system

**Figure 5:**
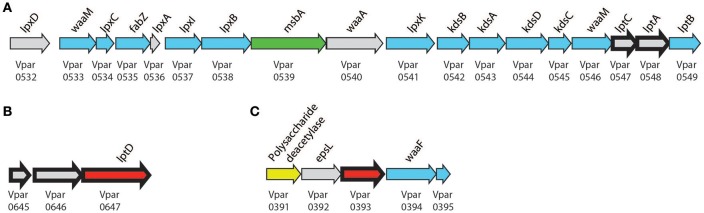
Lipopolysaccharide biosynthesis clusters. Biosynthesis clusters found within *V. parvula* of **(A)** core biosynthetic and transport machinery, **(B)** conserved *lptD* cluster, **(C)** proposed LPS modification cluster. Bold arrows represent peptides detected in the OM. Localization is presented by color: Gray, unclear; Blue, cytoplasmic; Green, IM; Yellow, Periplasmic; and Red, OM. Genomic coordinates are **(A)**, CP001820.1:650916–670828 (+); **(B)** CP001820.1:780878–784136 (+); **(C)**, CP001820.1:477425–481382 (+).

As we previously reported (Antunes et al., 2016), Negativicutes have the genetic potential to make and transport LPS to the OM. Most LPS biosynthesis genes are embedded in the diderm cluster in Negativicutes genomes, including that of *V. parvu* (Figure 5A). We were previously unable to identify the OM flippase components LptD or LptE, nor any component of potential O-antigen biosynthesis (Antunes et al., 2016). Here, an LptD homolog (Vpar_0647) was detected in the OM and SE fractions and identified as being an OM protein by localization prediction software (Figure 3, Table 1, and Table S1). This potential LptD encoding gene is located outside of the diderm cluster in a different region of the genome (Figure 5B). Interestingly, two proteins encoded by genes upstream of *lptD* (*vpar_0645* & *0646*) were both found in the OM and SE fractions (Figure 5B and Table S1). In addition, *vpar_0646* and *lptD* are conserved in sequence and synteny among all Negativicutes members (Table S3). We were unable to ascertain the function of Vpar_0645 & 0646 as we could find no conserved domains or clear homologs in sequence databases. It is possible that these proteins function with the Lpt complex of *V. parvula* or are functional equivalents of LptE, which caps the transport pore of LptD.

Finally, we detected the periplasmic components LptA (Vpar_0548) and LptC (Vpar_0547) in our OM and surface-exposed fractions. LptC was excluded from our final OM dataset because it did not pass the bioinformatics filter due to a lack of any discernible signal sequence. While we cannot exclude a contamination of periplasmic components in our OM fraction, it is possible that LptA and LptC were dragged to the OM fraction by strong interaction with LptD, thereby suggesting the existence of a complete Lpt complex in *V. parvula* similar to other studied bacteria (Villa et al., 2013).

### O-antigen in *V. parvula?*

Despite the presence of a complete pathway to make the lipid-A core of LPS, we previously could not predict if Negativicutes make O-antigen or not (Antunes et al., 2016), and the structure of *Veillonella'*s LPS is currently unknown. Here, we detected a protein in the OM fraction that may be used for further LPS processing (Vpar_0393). Although this protein has no conserved domains, we could tentatively infer its function based on the surrounding genomic region (Figure 5C). This region contains three genes probably involved in sugar anabolism (*vpar*_0391, 0392, 0394) and is well conserved only among the Negativicutes. The first protein, Vpar_0391, contains a CE4_SF domain; this domain is characteristic of polysaccharide deacetylases, such as PgaB or IcaB. The second protein, Vpar_0392, has the highest similarity to EpsL from a plasmid of *Lactococcus lactis*, which has no known function, yet is the final gene in the *eps* operon of exopolysaccharide biosynthesis (Forde and Fitzgerald, 2003). Although Vpar_0392 and EpsL are clear homologs, none of the surrounding genes bear any remarkable similarity. Vpar_0394 is annotated as WaaF, an enzyme that is known to be specific of diderm bacteria. It is responsible for O-antigen attachment to LPS by means of transferring a L-glycero-D-manno-heptose residue to the core oligosaccharide moiety of LPS. Mutation of this gene in *Salmonella* results in increase in sensitivity to antibiotics, detergents, and bile salts (Brooke and Valvano, 1996). Together, these data suggest that this could represent a new gene cluster responsible for O-antigen biosynthesis. To confirm this hypothesis and evaluate whether *V. parvula* LPS contains O-antigen we extracted and visualized its LPS on SDS PAGE (Figure S1). The extraction showed the characteristic ladder-banding pattern of O-antigen, however further characterization is necessary to determine *V. parvula* LPS structure and function.

### *V. parvula* has many potential surface exposed adhesins

One of the most prevalent and important aspects of *Veillonella*'s lifestyle is its ability to form biofilms and interact with host cells or other members of the microbiota where it has been identified. Such interactions are mediated by adhesins and *Veillonella* has many of them (Hughes et al., 1992); these adhesins represent prolonged adaptation and evolution to the biofilm niche of *Veillonella*. By controlling the type and quantity of adhesins expressed it could prefer one substrate or cell and bind with high affinity. The presence of eight putative hemagglutinins belonging to the YadA-like family of trimeric autotransporters was recently reported in *V. atypica* OK5, and one of them (Hag1) was shown to be responsible for co-aggregation with resident flora in the oral cavity (Zhou et al., 2015).

Among the 12 trimeric autotransporters containing a typical YadA anchor domain in *V. parvula* DSM 2008 (Figure S2), we found 10 in all three samples (Table 1). These 10 trimeric autotransporters possess typical Yad_Head, Yad_Stalk, and Cter Beta-barrel Yad _anchor domains. They are likely adhesins of the hemagglutinin family (Bassler et al., 2015). Strikingly, six of these 10 trimeric autotransporters are located in a massive gene cluster (56 kb) (Figures S2, S3A). It is difficult to ascertain if this cluster is conserved in all Negativicutes and how many trimeric autotransporters it contains, as most of these genomes are in scaffolds. Trimeric autotransporters contain many repeated regions, are modular in nature, contain many recent duplication events, and may get to be very large; as such, many Negativicutes scaffolds terminate in these genes thereby preventing synteny analysis. Two trimeric ATs are notable (Figure S2A): Vpar_0450 was not included in our final list of 78 OM proteins due to no detection in the OM fraction. It probably had too low of a concentration to be detected. Vpar_0045 is composed almost entirely of a Yad_anchor domain and thus is probably not functional and was not detected in any of our samples.

Interestingly, three trimeric autotransporter adhesins of *V. parvula* (Vpar_0041, Vpar_0100, Vpar_1664) possess a C-terminal SLH motif that is located downstream of the Beta-barrel YadA_anchor domain (Figure S2). This suggests a topology where the SLH domain is periplasmic and interacts with the peptidoglycan, similar to OmpM, to stabilize the trimeric configuration of these adhesins. We found this type of architecture between SLH and YadA anchor domains present in other autotransporters. However, it is restricted to the Negativicutes pointing to a unique feature potentially linked to their lifestyle (data not shown).

Upon further analysis, we noticed a distinct pattern of distribution for the presence of flagella and trimeric autotransporters in Negativicutes (Figure 4). The Veillonellaceae and Acidaminococcaceae contain YadA-like trimeric autotransporters and lack flagella, while the Selenomonadaceae and Sporomusaceae typically possess flagella and generally lack the YadA-like proteins, with only two exceptions (Figure 4). This peculiar distribution of flagella or adhesins between strains has not been observed previously and presents a unique case of evolution driven by lifestyle with the distinct presence of adhesion or motility. This observation provides a unique opportunity to study the transition between adhesion and motile life style, especially in *Mitsuokella multacida* DSM 20544, which represents an interesting specimen: it belongs to the Selenomonadaceae, which are typically flagellated, however it has lost its flagellum cluster and has acquired adhesins (Figure 4).

The *V. parvula* genome also contains two potentially functional filamentous hemagglutinin (Fha)-like proteins transported by the two-partner system (TPS) (Figure S2B): Vpar_0979/Vpar_0980 and Vpar_1413/Vpar_1414 as TpsA adhesin/TpsB transporter pair. We detected only the TpsA adhesin Vpar_1413 in the OM and SE fractions (Figure 3, Table 1, and Table S3). Its transporter Vpar_1414 was detected in the WC fraction but may have been present at too low concentration to be detected in the OM or SE fractions. The other system, Vpar_0979-Vpar_0980, was not detected in any fraction, including the whole protein fraction, suggesting an absence of its expression in our growing conditions. It may only be expressed upon contact with a host cell or when interacting with other microbial species. Furthermore, Vpar_0979 SEC secretion signal was only detected by one out of three programs and may represent a degenerate coding sequence.

In addition to trimeric and TPS systems that almost exclusively correspond to adhesins, *V. parvula* encodes classical autotransporters that contain a C-terminus β-barrel domain for insertion in the OM and an N-terminus passenger domain for function. Although some classical autotransporters are adhesins, within others the passenger domain may carry additional virulence functions such as protease or lipase domains (van Ulsen et al., 2014). We found four genes encoding such classical autotransporters within the genome (*vpar_0037, vpar_0298, vpar_0330, vpar_1322*) (Figure S2C). Vpar_0037 seems to be composed solely of a Beta-Barrel domain without any passenger domain or signal sequence; as such it is probably not functional. Vpar_1322 has a passenger domain with no detectable secondary or tertiary structural homology to known functional domains. Neither of these two autotransporters were detected in any of our samples suggesting they are not produced in our growth conditions. One that was found in the OM fraction, Vpar_0298, has a detectable homology to ShdA, the AidA-like adhesin of *Salmonella typhimurium*. Vpar_0330 is an autotransporter with no clear functional identification of its passenger domain, however we could find weak homology to a polysaccharide lyase-like protein using Phyre2 (see Section Materials and Methods); we detected Vpar_0330 both in our OM and SE fractions. Interestingly, *vpar_0330* was recently shown to be upregulated in *V. parvula* in a mouse tumor colonization model compared to *in vitro* growth, both with and without co-culture of *Pseudomonas aeruginosa* (Pustelny et al., 2015). Furthermore, it was highly upregulated in caries when compared to healthy teeth (Do et al., 2015). These data suggest that this autotransporter may be an important colonization factor in *Veillonella* and a priority target for further investigation.

### Other outer membrane features

#### Lipoproteins

Lipoproteins are found in most diderm bacteria and can be targeted to the outer membrane by the LOL transport machinery (Sutcliffe, 2010; Dowdell et al., 2017). We previously noted the absence of any LOL transport components (*lolA-E)* in any Negativicute genome (Antunes et al., 2016), however we find that 17 out of 78 OM proteins contain a lipoprotein signal sequence as predicted by LipoP and PRED-LIPO software (Table 1 and Table S3). To resolve this incongruence, we further analyzed the MS data of OM proteins that contained a cysteine residue. We searched for any mono, di, or triacylglycerol with a saturated C16 or C18 fatty acid as a modification (Data not shown). No such modification was found on any of the proteins, supporting the hypothesis that *Veillonella* does not have OM-targeted lipoproteins. These data are not conclusive though, as our MS analysis was not designed specifically for this purpose, and further studies should be performed. If *V. parvula* is shown to possess OM lipoproteins, it may use an alternative pathway, which remains to be identified. Another possibility is that these proteins may be tethered to the IM with a lipid moiety and span the periplasmic space in addition to being in the OM.

#### Cell ultrastructure

**Figure 6:**
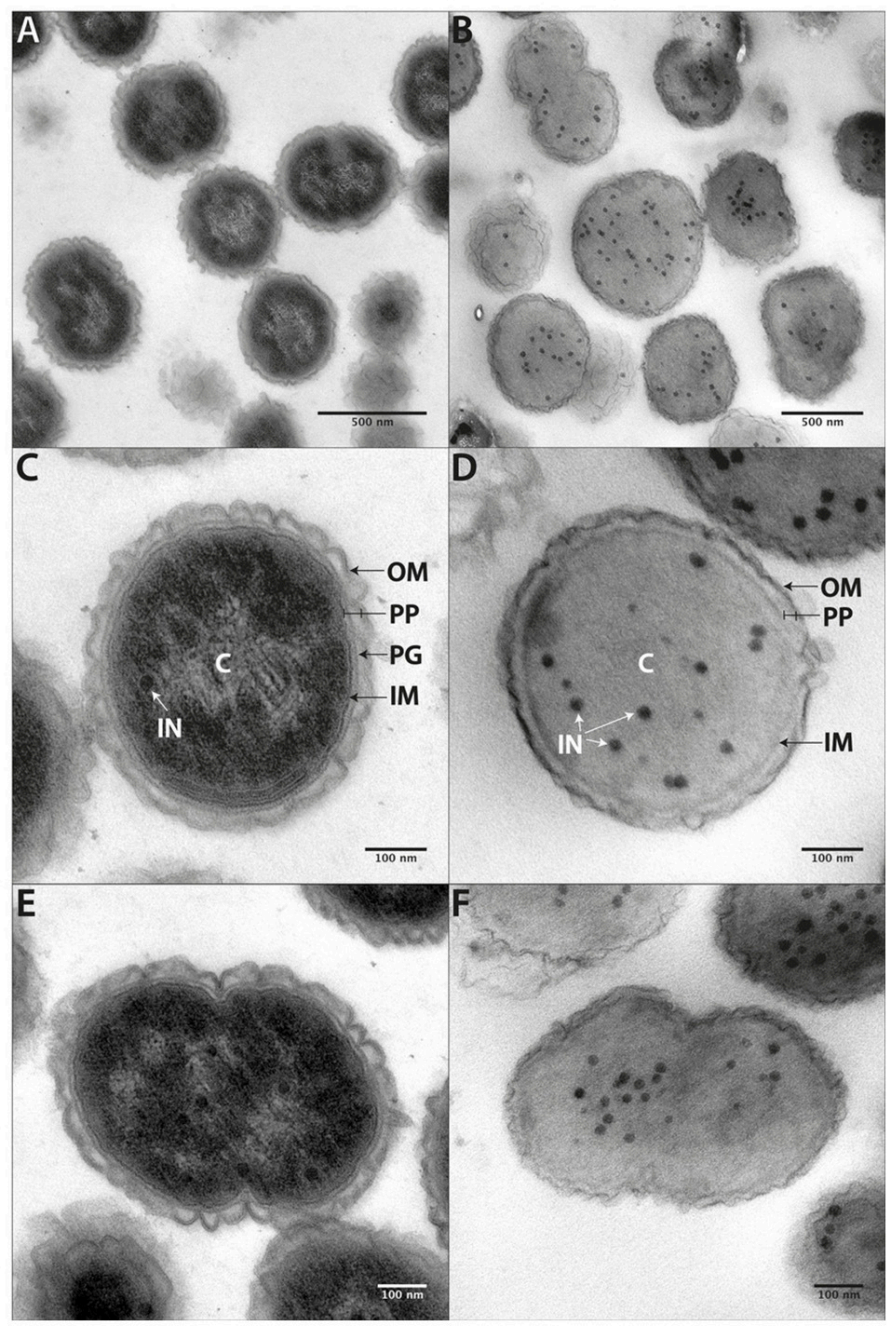
Cell ultrastructure. Ultrathin sections of chemically fixed **(A,C,E)** or high-pressure frozen **(B,D,F)** *V. parvula* cells. At higher magnification the inner membrane (IM), the peptidoglycan (PG) containing periplasm (PP), and the slightly waved outer membrane (OM) become apparent. In chemically fixed cells, an electron brighter area within the periplasm most likely represents DNA. Electron dense circular structures (IN) can be seen within the homogenous cytoplasm of high-pressure frozen cells and, to a less extent also in chemically fixed cells.

Although images of *Veillonella* are available in the literature, high quality electron microscopy has not been performed. We carried out ultrastructural analysis of *V. parvula* cells in two different ways: via chemical fixation (Figures 6A,C,E) and via high-pressure freezing of cells (Figures 6B,D,F). Subsequent resin embedding and ultrathin sectioning of *V. parvula* cells delivered a clear insight into the cell wall composition of this organism. Within the homogenous cytoplasm, electron dense particles are visible especially after high-pressure freezing (Figures 6B,D,F). In chemically fixed cells, an electron brighter area in the central part of the electron dense cytoplasm might show the DNA (genome). In both fixation variants, the cytoplasm is surrounded by the cytoplasmic membrane, which is again surrounded by a periplasm. Within this periplasm, peptidoglycan can sometimes be detected as electron dense line (Figure 6C). At the outermost part of the cell envelope, an outer membrane covers chemically fixed as well as high-pressure frozen cells. As the wavy appearance of the outer membrane is present after both fixation methods, this might either represent a special structural feature or a preparation artifact due to imperfect adjustment of the osmolarity of the post-fixation solutions to the original growth medium of the organism. The relative high thickness and in some cases fluffy appearance of the outer moiety of the outer membrane (Figure S4) is a strong indication for presence of LPS, although an S-layer cannot completely be ruled out (see below).

#### An S-layer in *V. parvula*?

Surface layer proteins (S-layer proteins) are important cellular factors, in both monoderms and diderms. They are assembled as paracrystalline mono-layers on the cell surface of bacteria and archaea, are attachment sites for enzymes and/or substrates, or participate in interactions with abiotic surfaces or bacteria (Sara and Sleytr, 2000; Gerbino et al., 2015). We found two proteins containing three SLH contiguous domains (Vpar_1653 & Vpar_1654) in our OM and SE fractions (Figure 3, Table 1, and Table S3). Such domain architecture can be found in typical S-layer glycoproteins yet may also be associated to other functional domains such as hydrolases. Unfortunately, no other functional domains could be detected in Vpar_1653 and Vpar_1654. A classical S-layer has not been documented in *Veillonella*. A fluffy outer leaflet of the outer membrane was often visible, especially in high-pressure frozen cells (Figure S4). This may show the S-layer on top of the outer membrane, however it is more likely that this structure represents the LPS moiety. It is unlikely that a complete S-layer is produced, as these proteins are normally extremely abundant, but were 100 fold less abundant than OmpM in the OM fraction (Table S5).

Interestingly, *vpar_1653* and *vpar_1654* genes are in cluster along with a third gene (*vpar_1655*), which was also found in our OM and SE fractions (Table 1, Table S3). This third gene does not code for an S-layer protein and has no obvious function or conserved domains, other than one of unknown function (DUF4163). We could not find this three-gene cluster outside of *Veillonella* (Table S3), suggesting that it may have a genus-specific function. Further functional analysis is required to determine if *Veillonella* utilizes this gene cluster to make a typical S-layer or if it is used for enzymatic activity, interaction with surfaces and/or biofilm formation.

#### TonB-dependent transport

The TonB system is an OM transport mechanism present in most diderm bacteria, which has never been described in any Firmicute. Bacteria generally contain multiple TonB transport systems specifically associated with the acquisition of small molecules including vitamin B12 or metals such as iron (Krewulak and Vogel, 2011). We identified nine such TonB systems in the *V. parvula* genome, of which six were present in the OM and SE fractions (Figure 3, Table 1, and Table S3). Some of these systems must be functional to provide iron for the *V. parvula* haem cluster which has recently been characterized in *V. atypica* (Zhou et al., 2016). Interestingly, almost all of the operons encoding these TonB systems are located within a single genomic cluster downstream of the previously mentioned adhesion cluster (Figure S3B). Two other *tonB* operons are located at distant loci but were not found in our samples; specific molecules that were not present in our growing conditions might induce their expression.

#### Efflux pumps

TolC is an outer membrane channel responsible for export of antibiotics and other toxic compounds from the cell. These systems are important in antibiotic resistance and in infection (Zgurskaya et al., 2011). Among the two TolC-ABC transporters and three similar RND type efflux pumps that are encoded in the genome of *V. parvula* DSM2008, we detected three in our OM and SE fractions (Vpar_0525, Vpar_1003, Vpar_1367) and two in only the OM fraction (Vpar_0011, Vpar_1641) (Figure 3, Table 1, and Tables S1, S3). Intriguingly, it was previously shown that the RND pump Vpar_1367 is upregulated in an infectious community responsible for caries (Do et al., 2015), pointing to the importance of such systems to scavenge essential molecules in *in vivo* conditions in a multispecies context.

### Putative OM proteins of unknown function

**Figure 7:**
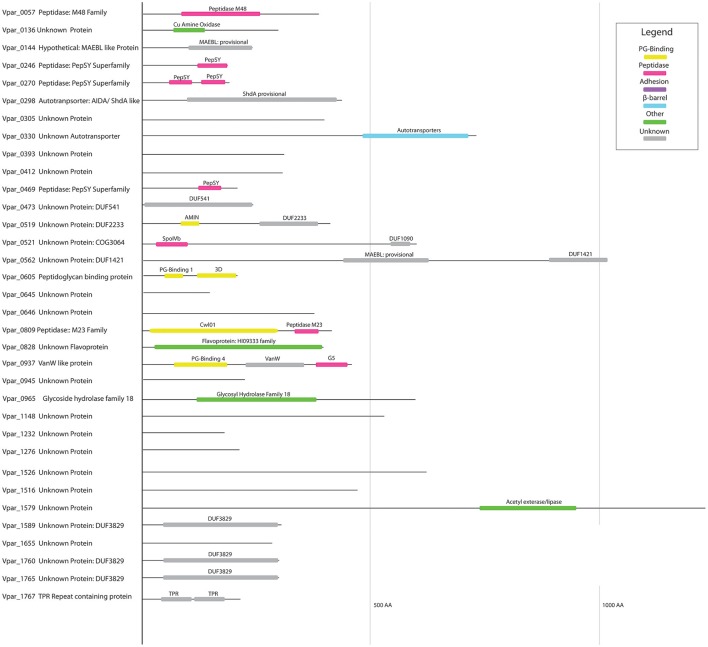
Domain structure of unknown OM proteins. Protein domains identified in all poorly annotated OM proteins at the NCBI CDD database, and colored according to proposed function based on CDD annotation.

Among the list of 78 OM proteins, 28 proteins have no clear function (Figure 3, Table 1, and Table S3), many of which may correspond to previously undescribed systems and novel OM-related functions. We represented these unknown function proteins, as well as six unknown proteins previously described in other sections, with their detected functional domains in Figure 7.

Of special interest is Vpar_0521 (COG3064). We previously found it is well-conserved within the diderm Firmicute, and hypothesized its possible involvement in a yet unknown OM-related function (Antunes et al., 2016). Vpar_0521 contains two domains (Figure 7): an N-terminus SpoIVB and a C-terminal DUF1090. The N-terminus domain, SpoIVB, is an autoprotease (S5 peptidase) involved in sporulation in the Firmicutes; it is normally synthesized in the forespore and transported to the interspace between the forespore membrane and the outer spore membrane, where it functions in signal transduction (Campo and Rudner, 2007). Because *V. parvula* is a non-sporulating Firmicute, the role of this SpoIVB domain remains to be determined. The other domain, DUF1090, is an uncharacterized domain found in all three domains of life, and predominantly present in Bacteria. For example, the *E. coli* YqjC protein only contains this domain. Only one study has been performed on the corresponding gene and it was found to be down regulated when the sigma factor *rpoS* gene was mutated (Zhang et al., 2012). In the Stepdb database that details the localization of all *E. coli* proteins (Orfanoudaki and Economou, 2014), YqjC is annotated as periplasmic. In some bacteria, this domain constitutes the C-terminal hydrophobic substrate-binding domain of the chaperone DnaK and it may have a similar function in Vpar_0521. These indications, and the conservation of Vpar_0521 in diderm Firmicutes, make this protein an important target of future study.

We found three proteins corresponding to the DUF3829 domain (*Vpar_1589, Vpar_1760, Vpar_1765*) (Figure 7). Although this domain is found in the distantly related Proteobacteria using a CDD search, the only close BLAST hits were from human-associated Negativicutes (Figure 4), more specifically the *Dialister, Centipeda, Selenomonas*, and *Veillonella*. We could not detect any homologs in *V. ratti* or any of the other rodents-associated *Veillonella* (Whitman, 2011). These unstudied proteins may be involved with interactions in the human niche, both for pathogenic and commensal lifestyles.

Three proteins contain one (Vpar_0246, Vpar_0469, Vpar_1597) or two (Vpar_0270) PepSY domains, and no other conserved domains. All proteins containing a PepSY domain were found in all three extractions except for Vpar_1597, which was not detected in any sample. This domain has not been extensively studied, yet it is known to possess a peptidase inhibitory function and is found throughout bacteria and some archaeal species (Yeats et al., 2004). The dominantly studied member of this family is YpeB, an inhibitor of the spore cortex lytic enzyme SleB in sporulating Firmicutes (Yeats et al., 2004). The four *V. parvula* OM PepSY proteins may function similarly to YpeB and inhibit peptidases of the M4 family or possess some unknown function.

## Conclusions

In recent years we have learnt how important *Veillonella* is to the human microbiome, infection, and immune development (Whitman, 2011; Arrieta et al., 2015; Hirai et al., 2016). Moreover, these bacteria deserve to be studied not only for these characteristics, but also for the evolutionary questions they pose. This work represents the first proteomic characterization of a diderm Firmicute cell envelope and provides important information to guide further characterization. It confirms that the Negativicutes cell envelope has many aspects of the classical and well-studied Proteobacterial Gram-negative cell envelopes, such as LPS, TolC, OmpA, and other components, yet it also possesses many unique and potentially ancestral characteristics, such as the peculiar OM-PG attachment system, the BAM/TAM complex, as well as potentially new OM systems. The function of these systems needs to be further characterized through mutational and expression studies. Finally, our results are instrumental in increasing our understanding of the lifestyle of *Veillonella*, including its potential for biofilm formation and its role in infection and host-interaction.

## Author contributions

DP prepared all samples and carried out bioinformatics analysis of genomic and proteomic data. MD, VH, and MM carried out mass spectrometry analysis. JF and AK carried out electron microscopy. CB supervised the study and analyzed the data. SG conceived and supervised the study, and analyzed the data. All authors participated in writing the manuscript.

### Conflict of interest statement

The authors declare that the research was conducted in the absence of any commercial or financial relationships that could be construed as a potential conflict of interest.

## Supplementary material

Figure S1 Glycostain of LPS. Thirteen and seventeen percentage SDS-PAGE loaded with identical samples and visualized with Pro-Q emerald glycostain. O-antigen producing positive control *E. coli* were loaded in lanes 1 and 3, while O-antigen negative extractions were loaded in lanes 2–4. Three biological replicates of *V. parvula* were loaded in lanes 5–7.

Figure S2 Adhesins domain structure. Figure demonstrating the domain structure of all trimeric autotransporters **(A)**, two partner systems **(B)**, and autotransporters **(C)**.

Figure S3 Trimeric autotransporter **(A)** and TonB **(B)** genomic clusters. Bold arrows represent peptides detected in the OM. Localization is presented by color: Gray, Unclear; Blue, Cytoplasmic; Green, IM; Yellow, Periplasmic; Purple, Secreted; and Red, OM.

Figure S4 High-pressure frozen cell illustrating LPS. The ultrathin section of a high-pressure frozen cell shows the cell wall of *V. parvula* **(A)**. Beside the inner membrane (IM), the outer membrane (OM), and the peptidoglycan (PG) within the periplasm, the fluffy outer leaflet of the outer membrane is visible at higher magnification **(B)**. This might represent the LPS moiety. Bold arrows represent peptides detected in the OM.

Table S1 Localization prediction and Mass spectrometry results for all proteins of *V. parvula* DSM 2008. Proteins predicted to be in the OM are in red.

Table S2 Localization prediction and Mass spectrometry results for 129 potential OM proteins of *V. parvula* DSM 2008.

Table S3 Localization prediction and Mass spectrometry results for 78 OM proteins of *V. parvula* DSM 2008.

Table S4 Type 4 pili components *in silico* digestion and detection.

Table S5 OM extraction gel excision Mass spectrometry results. Top 5 hits for each band are shown; Proteins predicted to be in the OM are highlighted.

Table S6 IBAQ results for the OM extraction.
