# Supplementary material for: Outer Membrane Proteome of Veillonella parvula: A Diderm Firmicute of the Human Microbiome
Source: Front Microbiol. 2017 Jun 30;8:1215. doi: 10.3389/fmicb.2017.01215 (PMC5491611; doi:10.3389/fmicb.2017.01215)
Supplement: Figure S1 — Glycostain of LPS. Thirteen and seventeen percentage SDS-PAGE loaded with identical samples and visualized with Pro-Q emerald glycostain. O-antigen producing positive control E. coli were loaded in lanes 1 and 3, while O-antigen negative extractions were loaded in lanes 2–4. Three biological replicates of V. parvula were loaded in lanes 5–7. [file Image1.PDF]

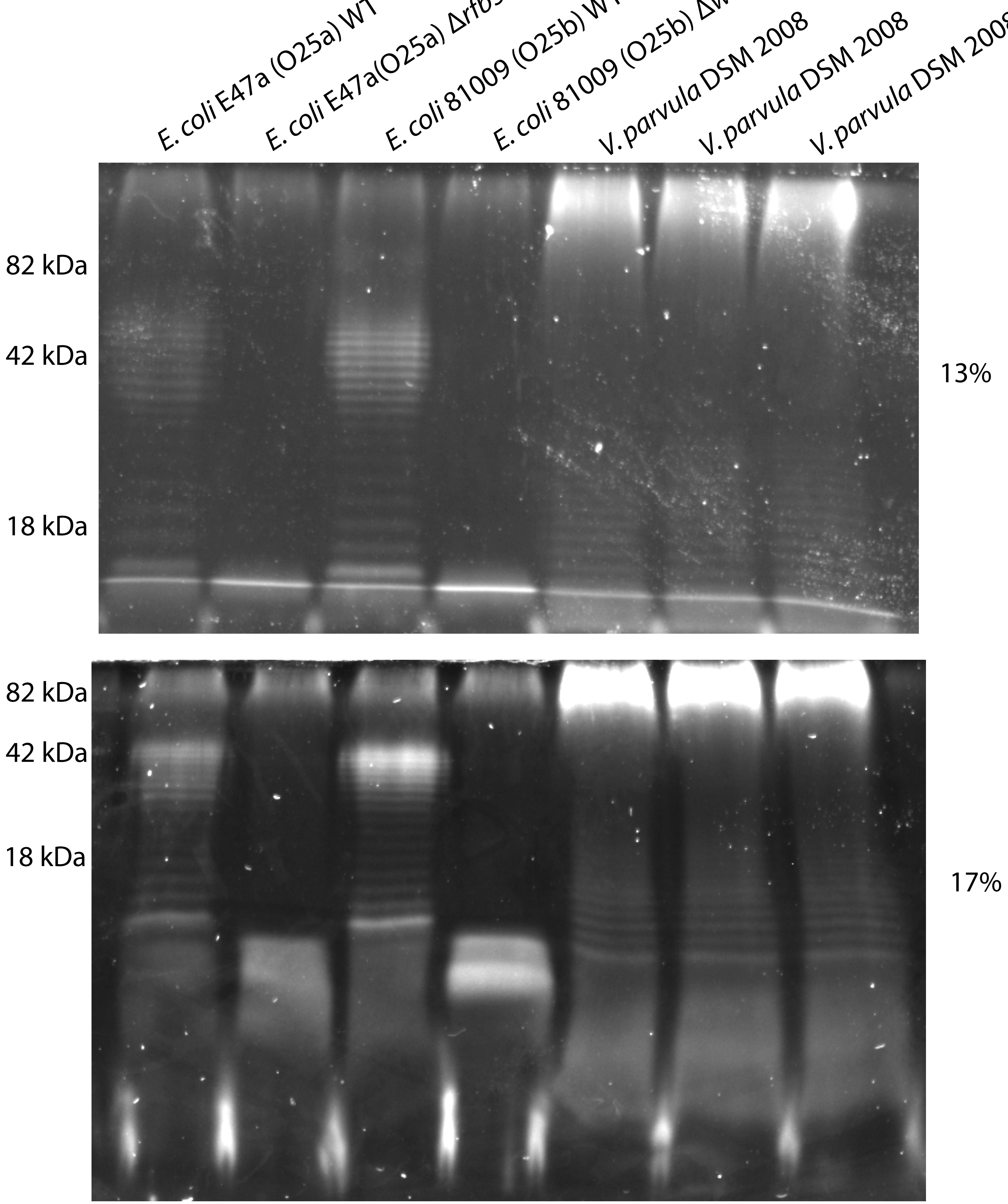

E. coli E47a (O25a) WT
E. coli E47a(O25a) Δ*rfb3*
E. coli 81009 (O25b) WT
E. coli 81009 (O25b) Δ*waaF*
V. parvula DSM 2008
V. parvula DSM 2008
V. parvula DSM 2008
82 kDa
42 kDa
18 kDa
13%
82 kDa
42 kDa
18 kDa
17%

Figure S1
