# Supplementary figures and images for: Outer Membrane Proteome of Veillonella parvula: A Diderm Firmicute of the Human Microbiome
Source: Front Microbiol. 2017 Jun 30;8:1215. doi: 10.3389/fmicb.2017.01215 (PMC5491611; doi:10.3389/fmicb.2017.01215)

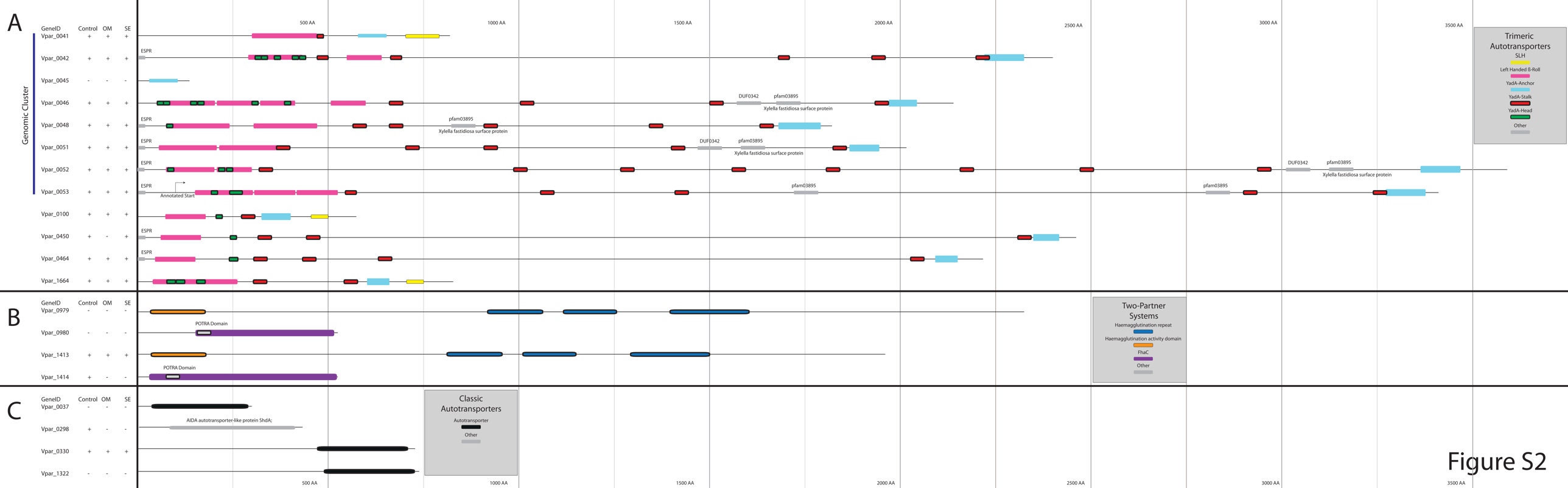

Supplement: Figure S2 — Adhesins domain structure. Figure demonstrating the domain structure of all trimeric autotransporters (A), two partner systems (B), and autotransporters (C). [file Image2.PDF]

A

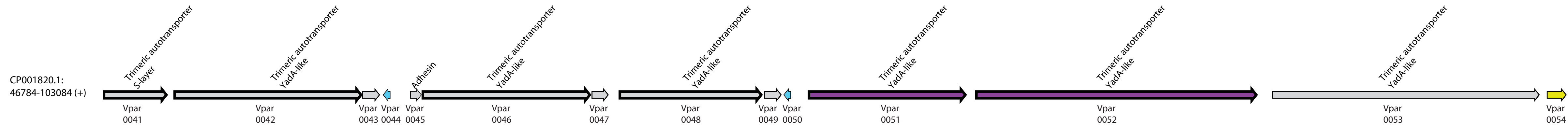

B

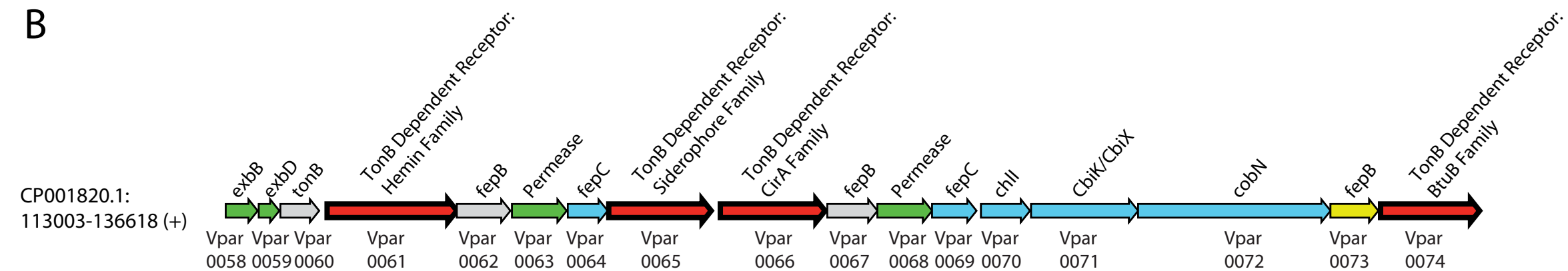

Figure S3

Supplement: Figure S3 — Trimeric autotransporter (A) and TonB (B) genomic clusters. Bold arrows represent peptides detected in the OM. Localization is presented by color: Gray, Unclear; Blue, Cytoplasmic; Green, IM; Yellow, Periplasmic; Purple, Secreted; and Red, OM. [file Image3.PDF]

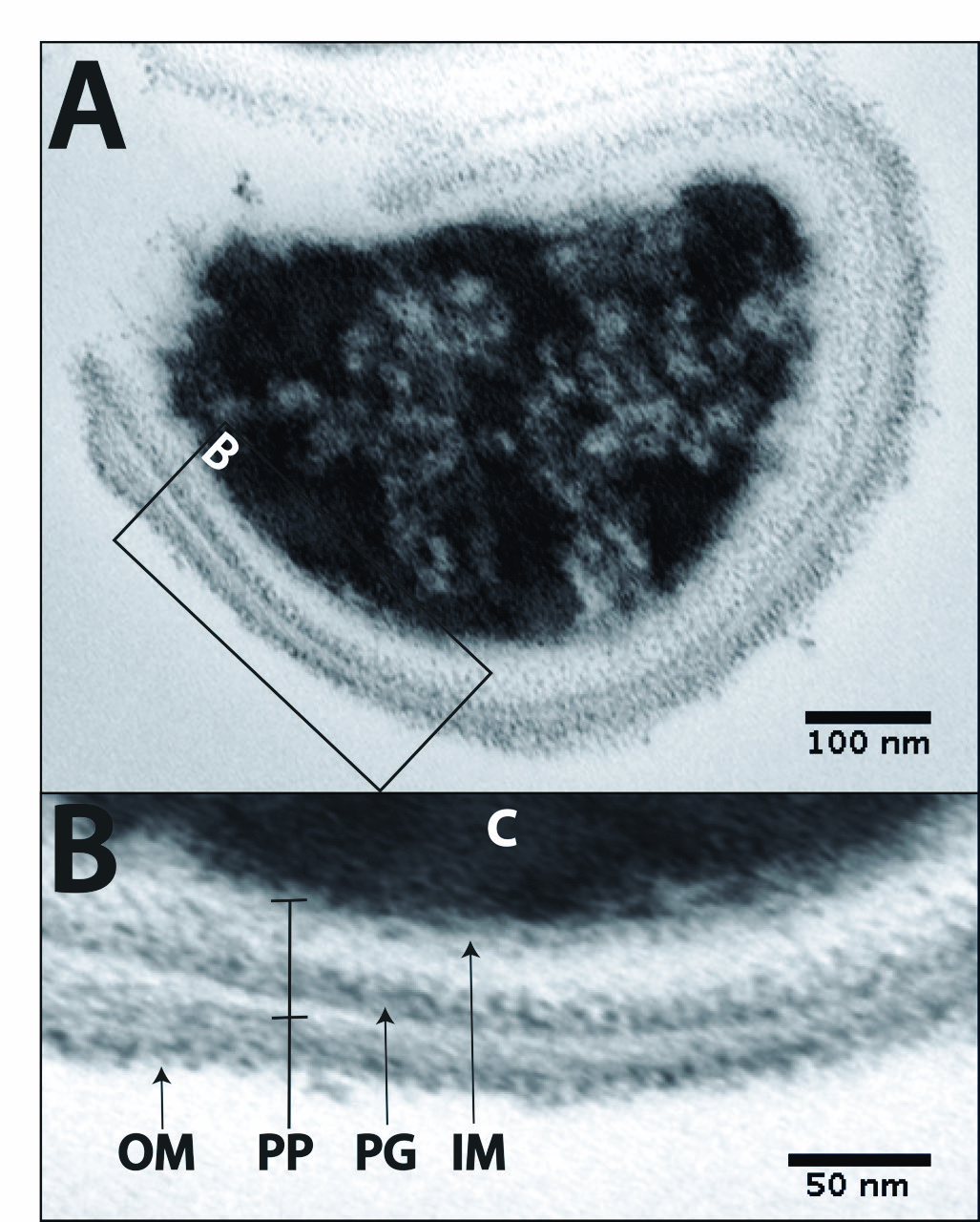

Supplement: Figure S4 — High-pressure frozen cell illustrating LPS. The ultrathin section of a high-pressure frozen cell shows the cell wall of V. parvula (A). Beside the inner membrane (IM), the outer membrane (OM), and the peptidoglycan (PG) within the periplasm, the fluffy outer leaflet of the outer membrane is visible at higher magnification (B). This might represent the LPS moiety. Bold arrows represent peptides detected in the OM. [file Image4.JPEG]

**Mean Intensity per Peptide (in log2)**

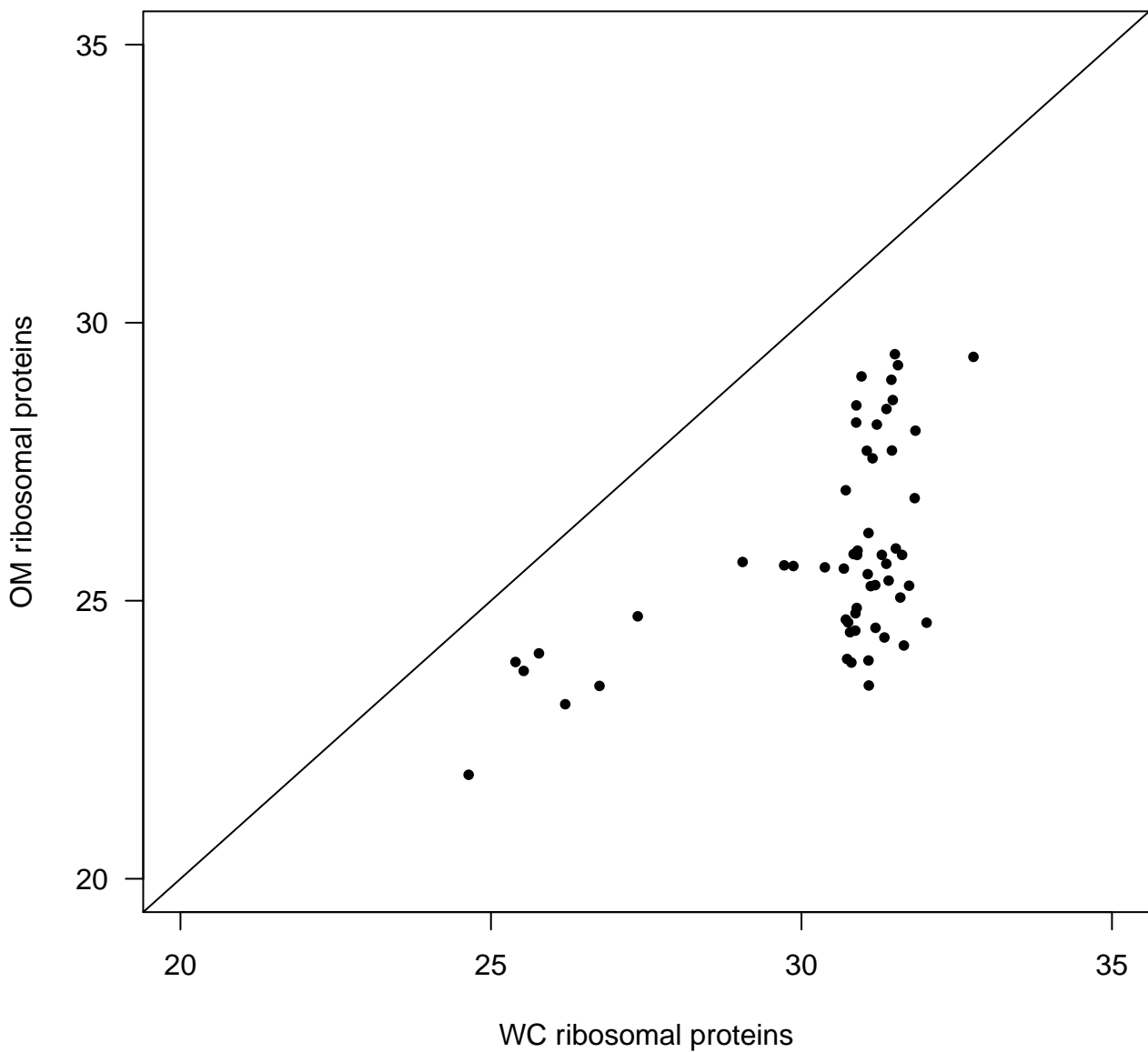

Supplement: Supplementary file 11 [file DataSheet1.ZIP › SupplementalFiles/ProtRibosomal_OMversusControlsamples.pdf]

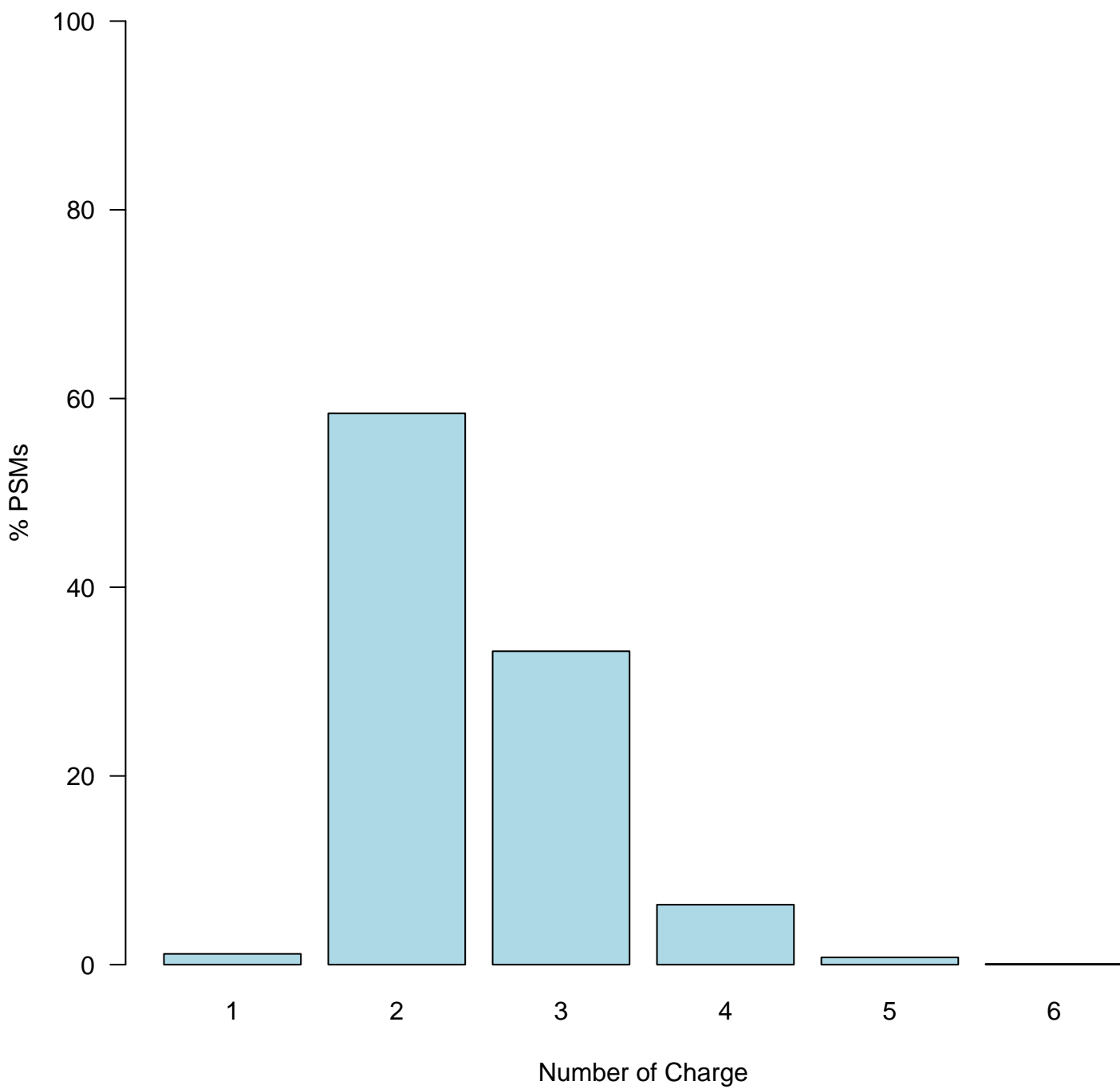

Supplement: Supplementary file 11 [file DataSheet1.ZIP › SupplementalFiles/PSMChargesPourcent.pdf]
