# Supplementary material for: Outer Membrane Proteome of Veillonella parvula: A Diderm Firmicute of the Human Microbiome
Source: Front Microbiol. 2017 Jun 30;8:1215. doi: 10.3389/fmicb.2017.01215 (PMC5491611; doi:10.3389/fmicb.2017.01215)
Supplement: Supplementary file 11 [file DataSheet1.ZIP › SupplementalFiles/Differential_Analysis_ Condition 1 _vs_ Condition 2_Daniel (1).pptx]

## Slide 1
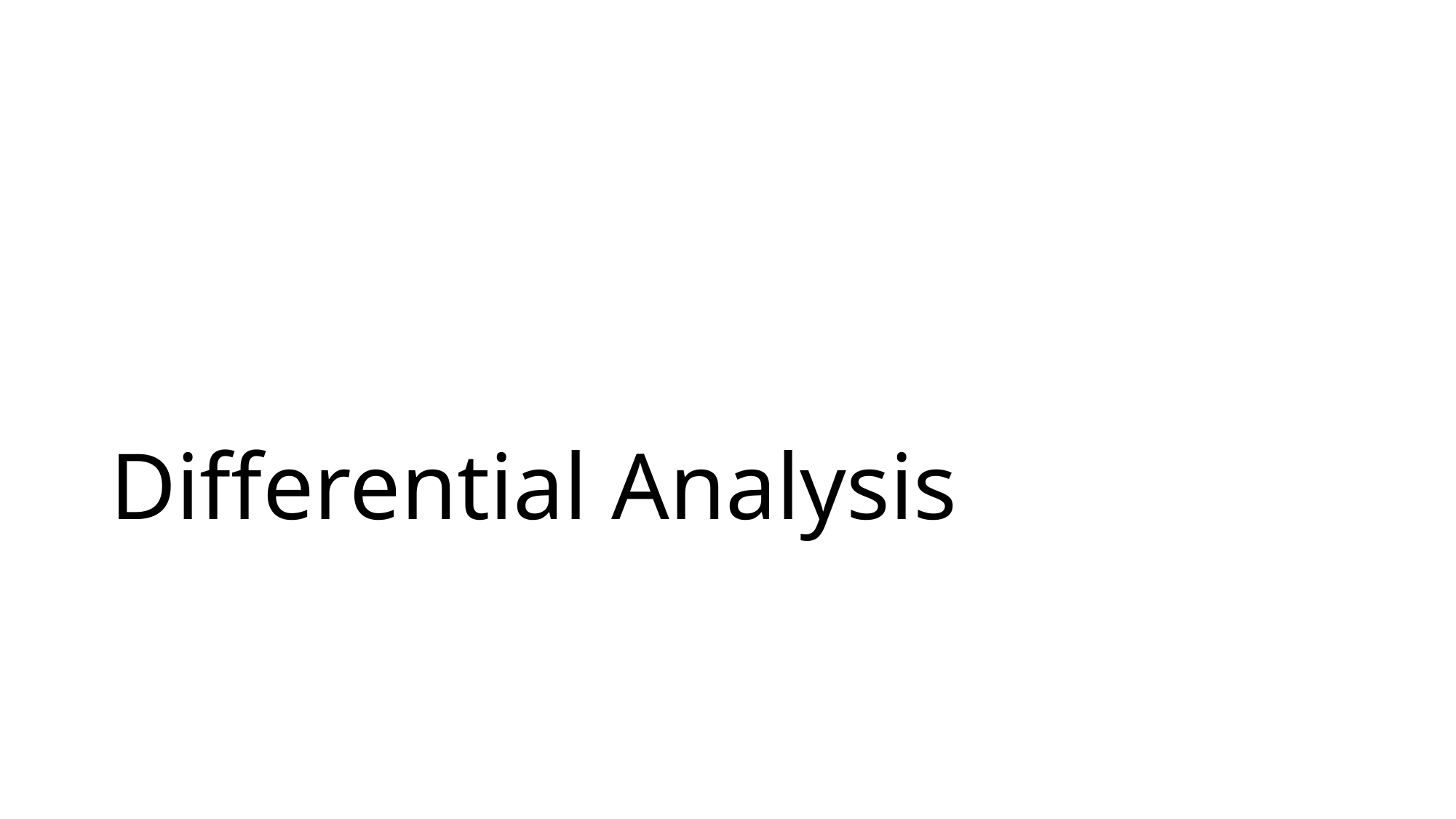

# Differential Analysis

## Slide 2
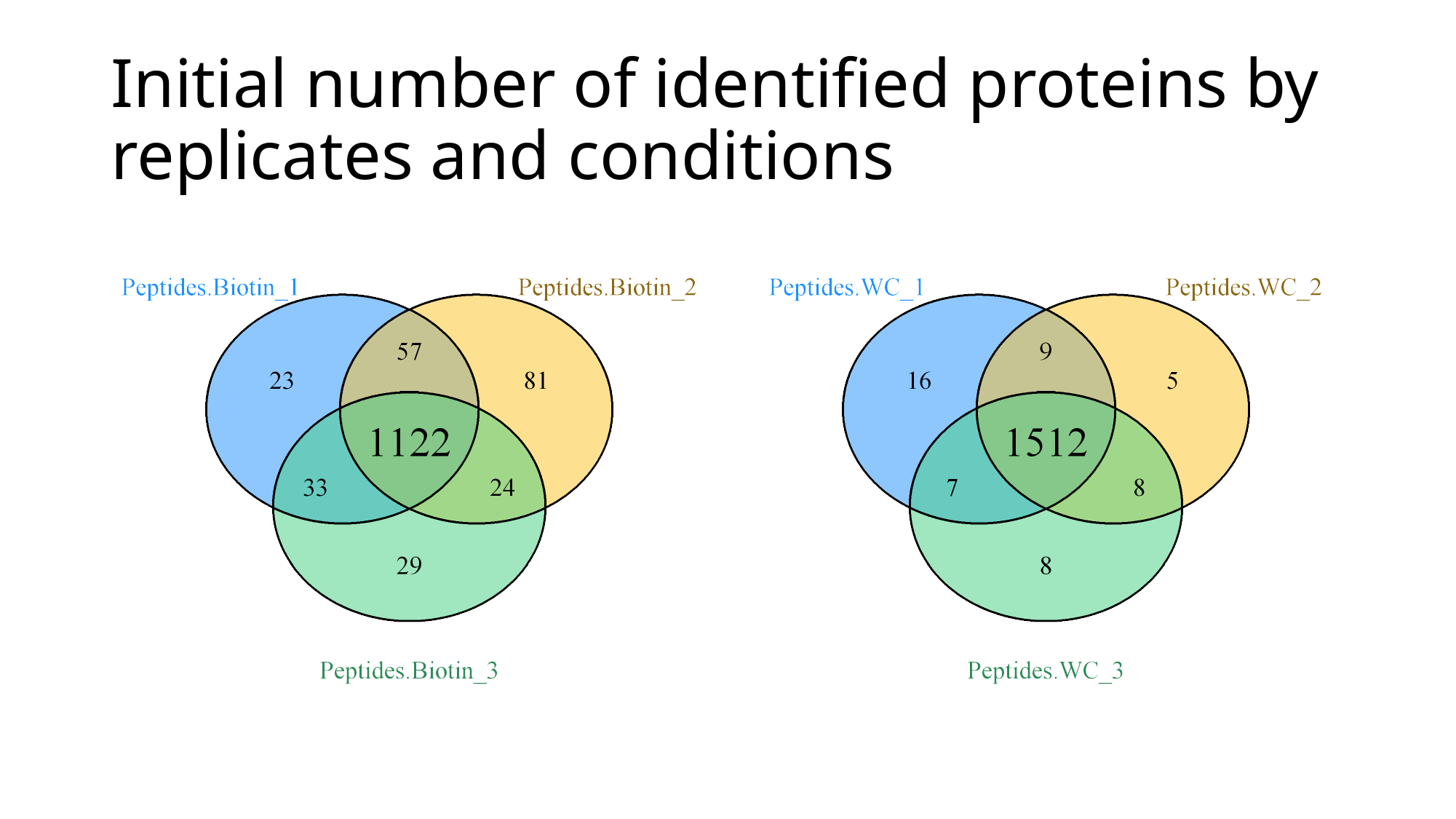

# Initial number of identified proteins by replicates and conditions

## Slide 3
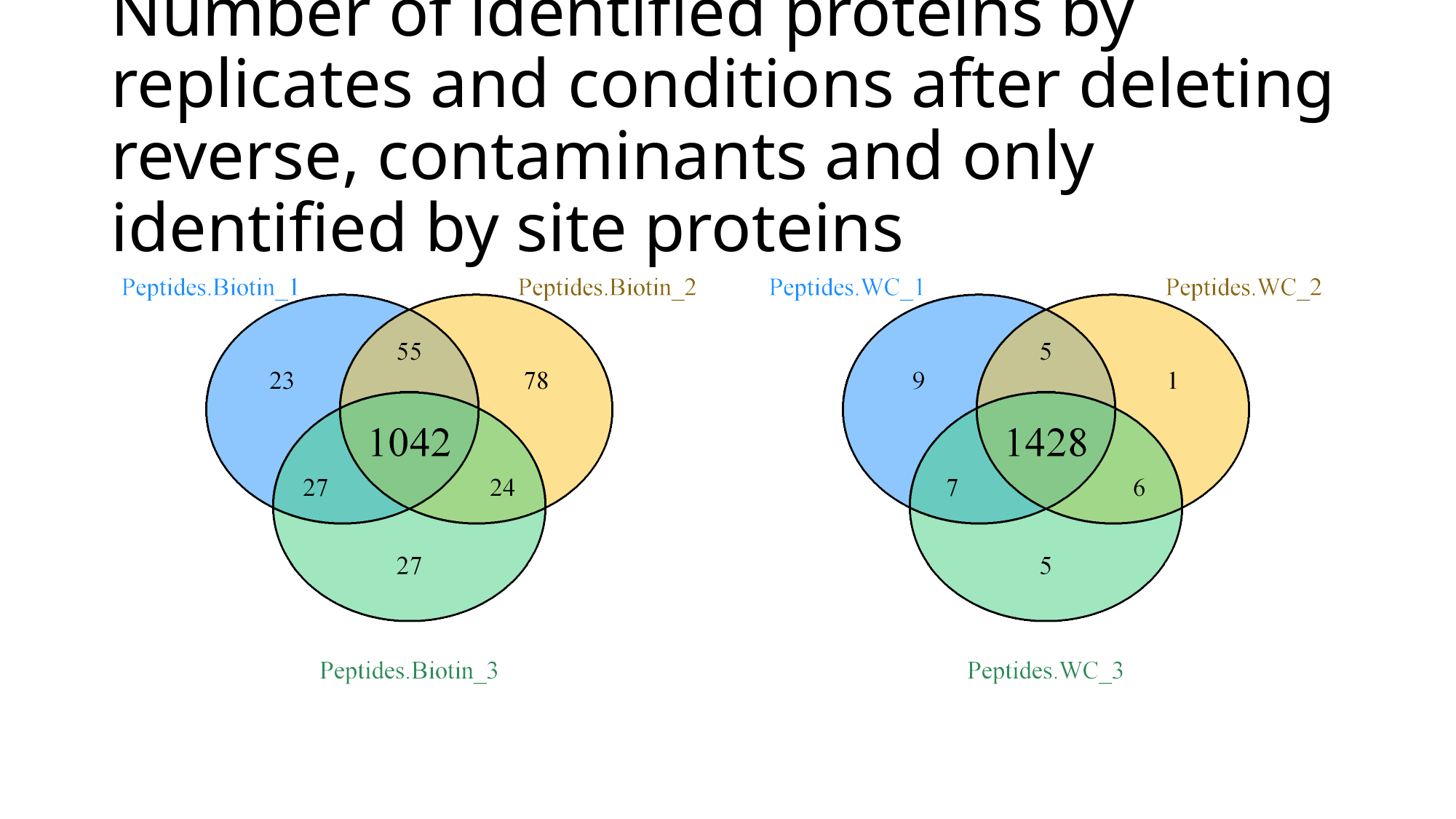

# Number of identified proteins by replicates and conditions after deleting reverse, contaminants and only identified by site proteins

## Slide 4
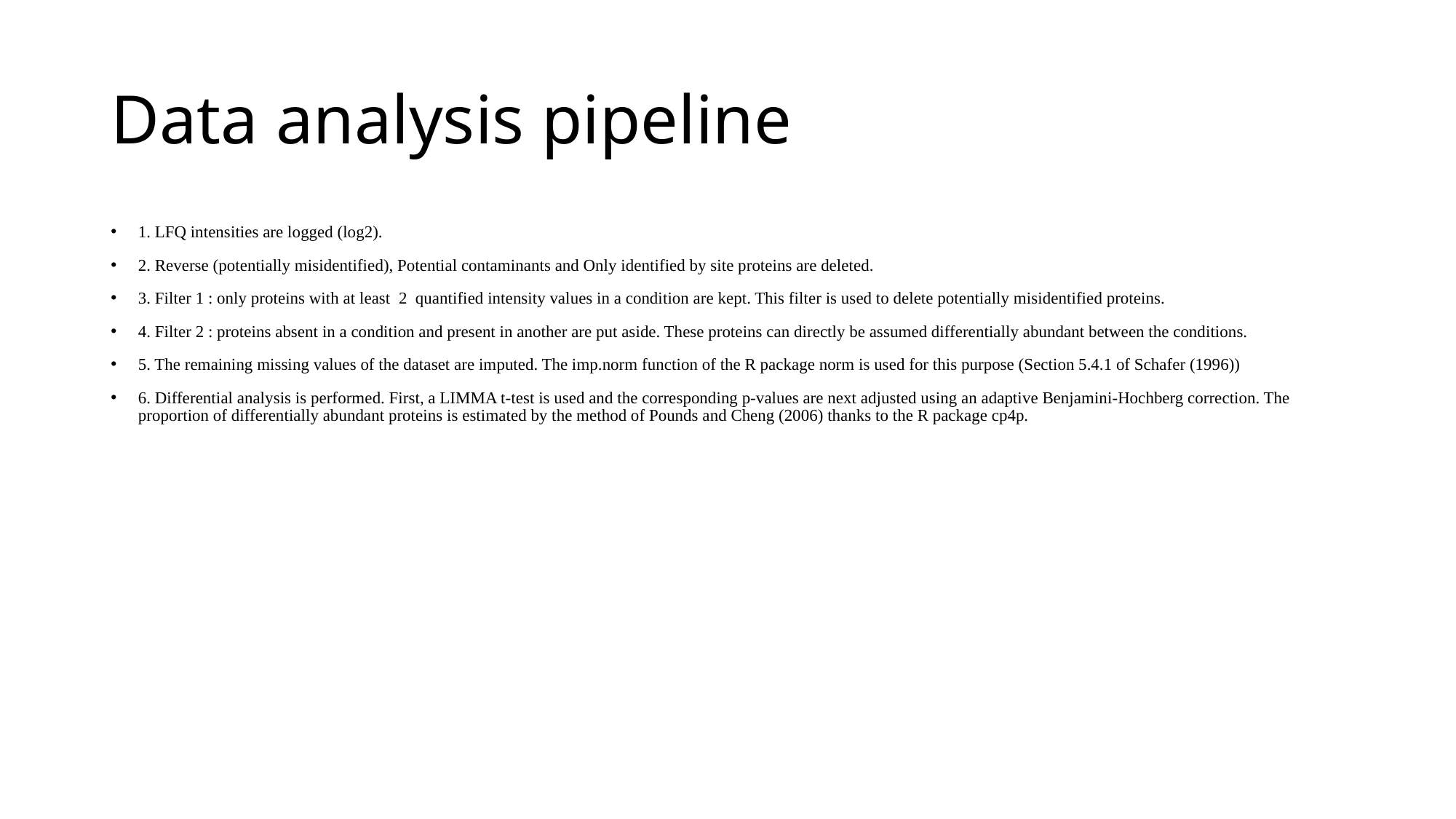

# Data analysis pipeline
1. LFQ intensities are logged (log2).
2. Reverse (potentially misidentified), Potential contaminants and Only identified by site proteins are deleted.
3. Filter 1 : only proteins with at least 2 quantified intensity values in a condition are kept. This filter is used to delete potentially misidentified proteins.
4. Filter 2 : proteins absent in a condition and present in another are put aside. These proteins can directly be assumed differentially abundant between the conditions.
5. The remaining missing values of the dataset are imputed. The imp.norm function of the R package norm is used for this purpose (Section 5.4.1 of Schafer (1996))
6. Differential analysis is performed. First, a LIMMA t-test is used and the corresponding p-values are next adjusted using an adaptive Benjamini-Hochberg correction. The proportion of differentially abundant proteins is estimated by the method of Pounds and Cheng (2006) thanks to the R package cp4p.

## Slide 5
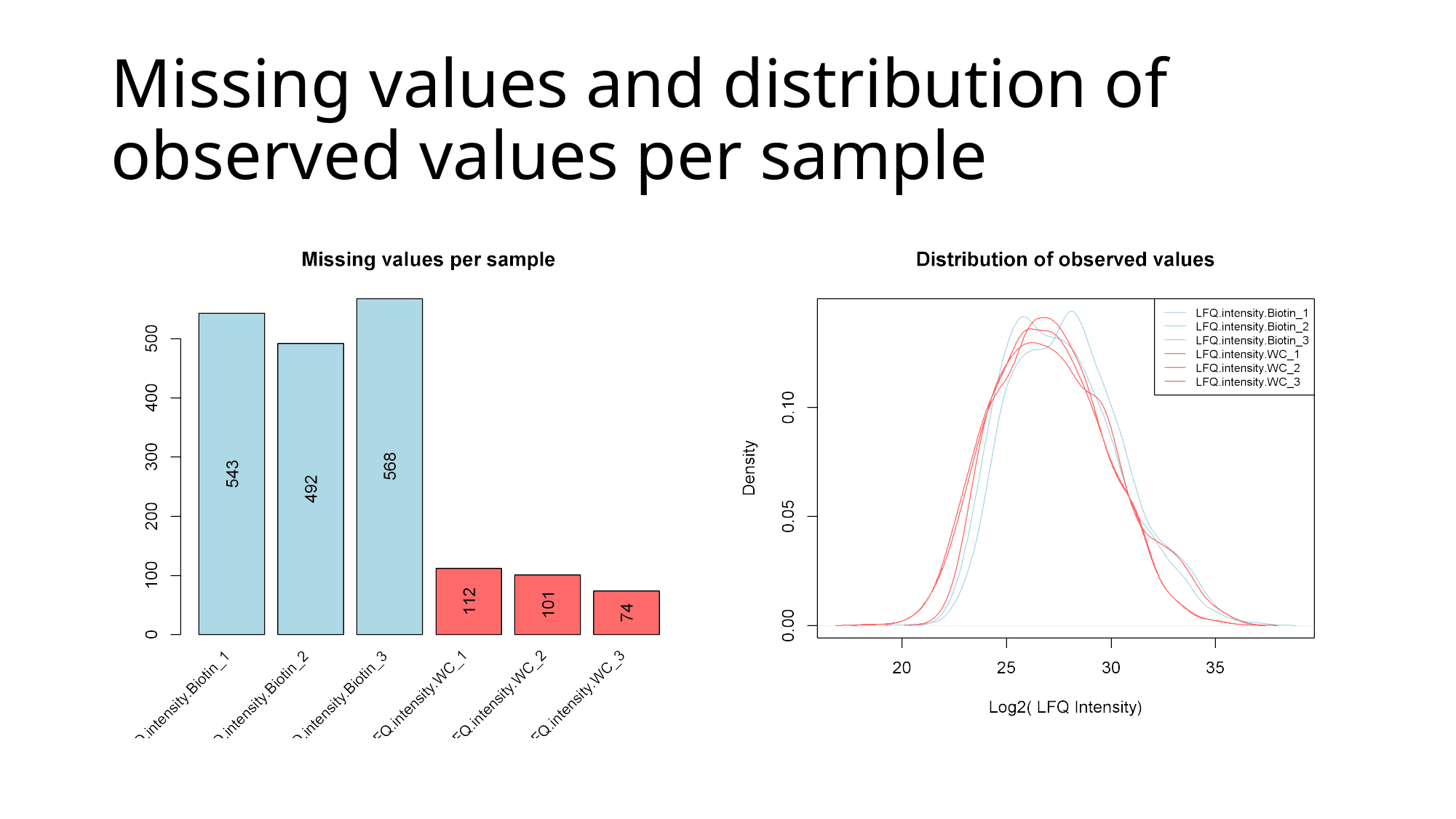

# Missing values and distribution of observed values per sample

## Slide 6
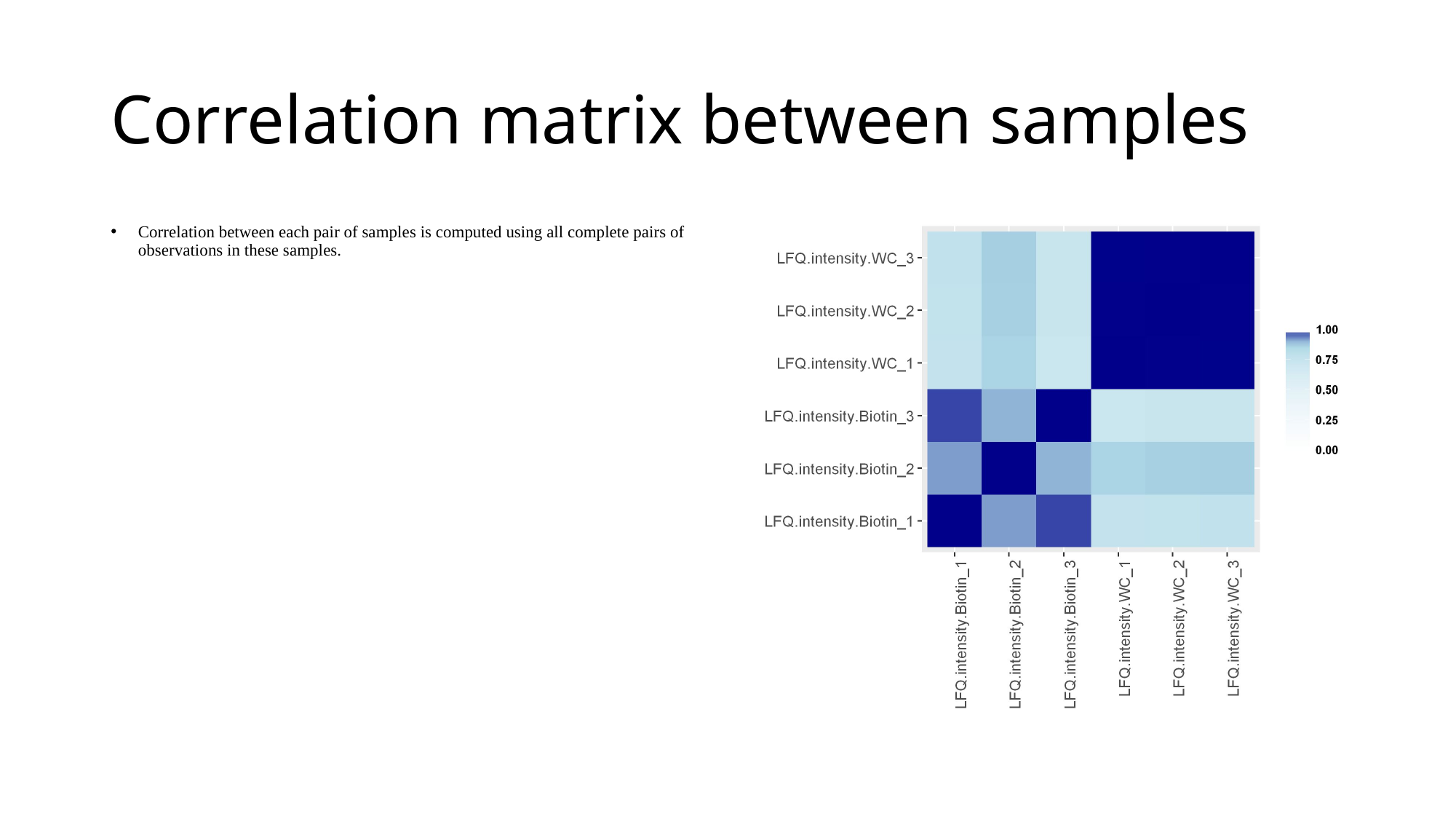

# Correlation matrix between samples
Correlation between each pair of samples is computed using all complete pairs of observations in these samples.

## Slide 7
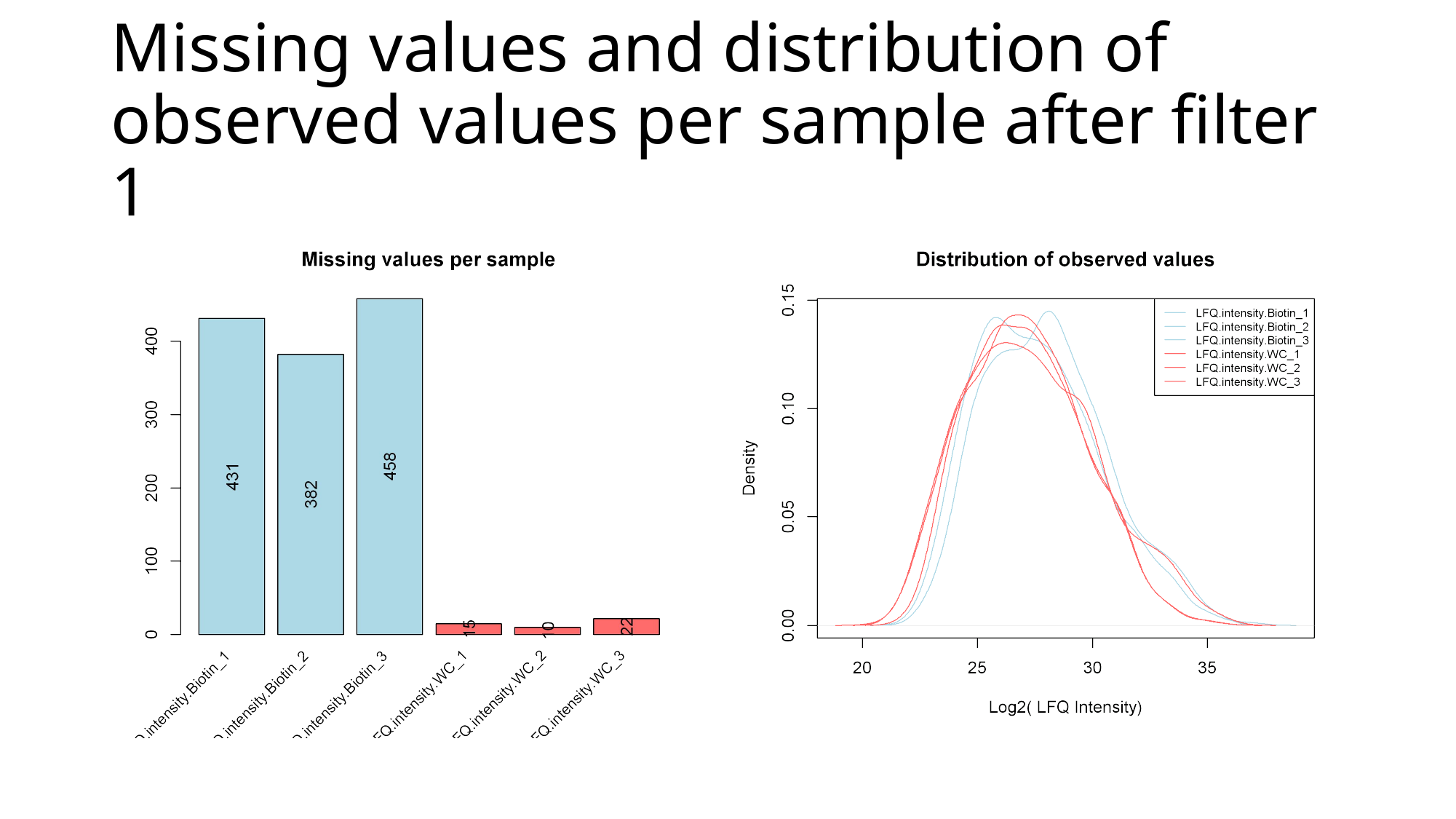

# Missing values and distribution of observed values per sample after filter 1

## Slide 8
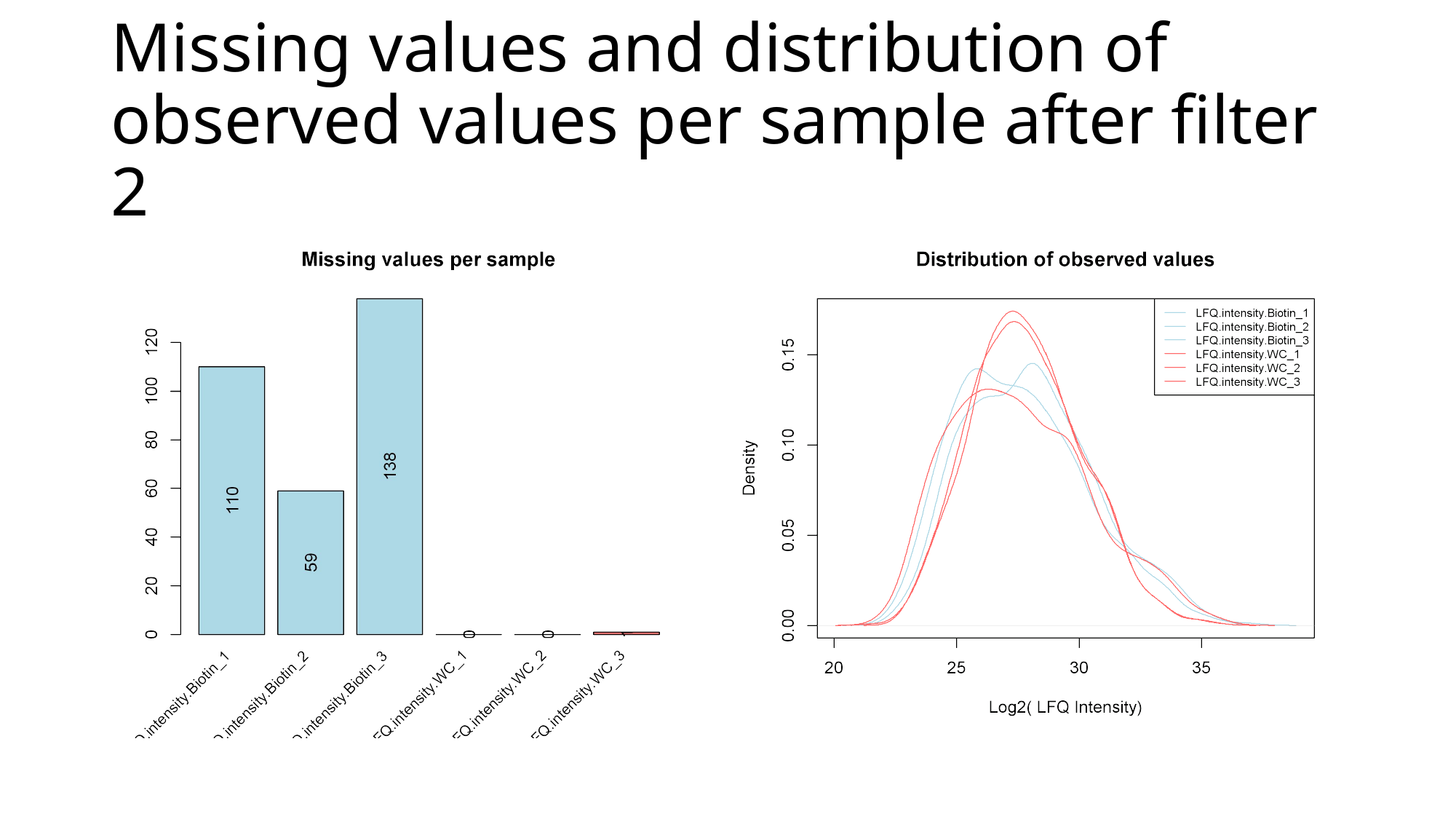

# Missing values and distribution of observed values per sample after filter 2

## Slide 9
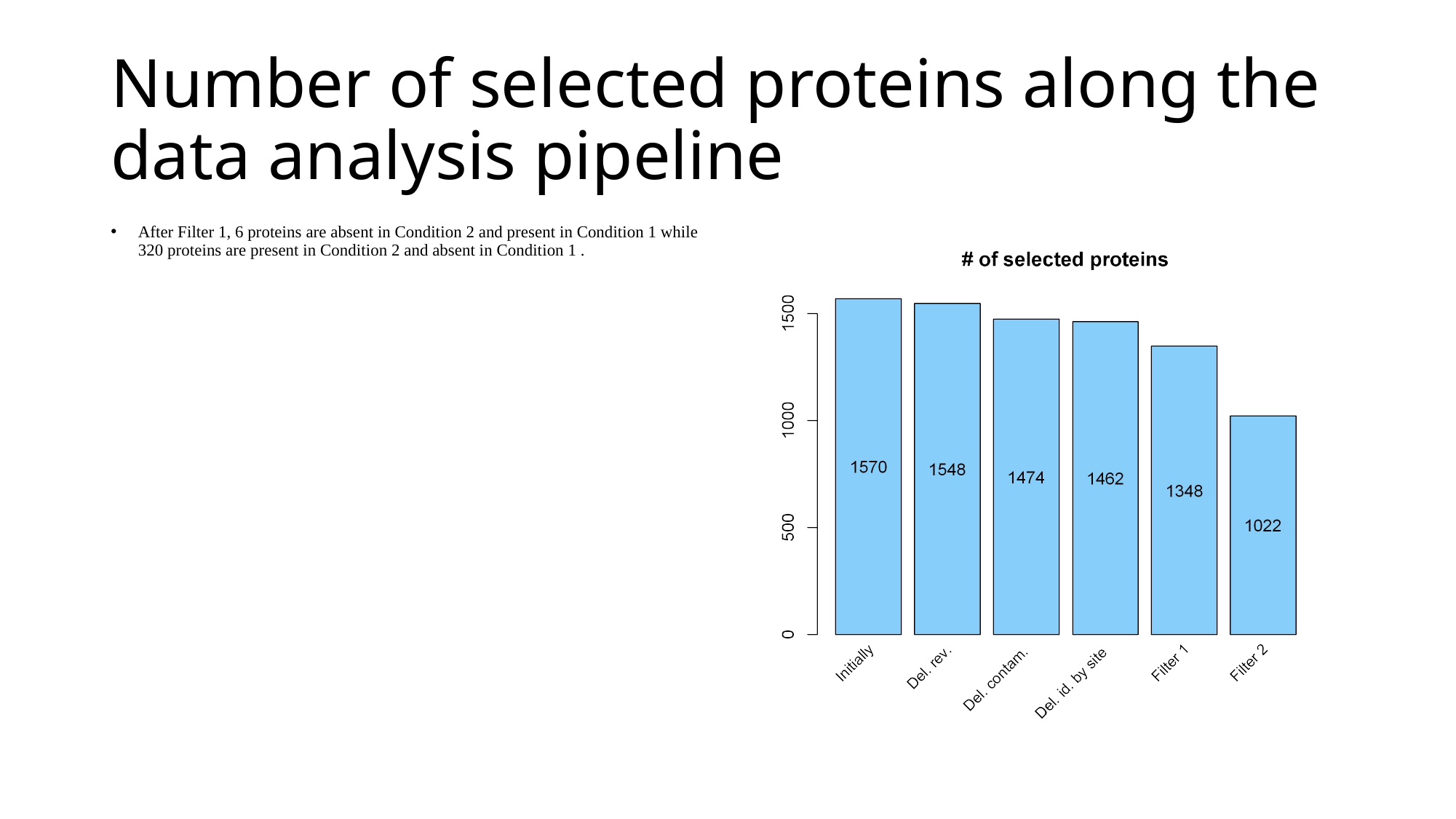

# Number of selected proteins along the data analysis pipeline
After Filter 1, 6 proteins are absent in Condition 2 and present in Condition 1 while 320 proteins are present in Condition 2 and absent in Condition 1 .

## Slide 10
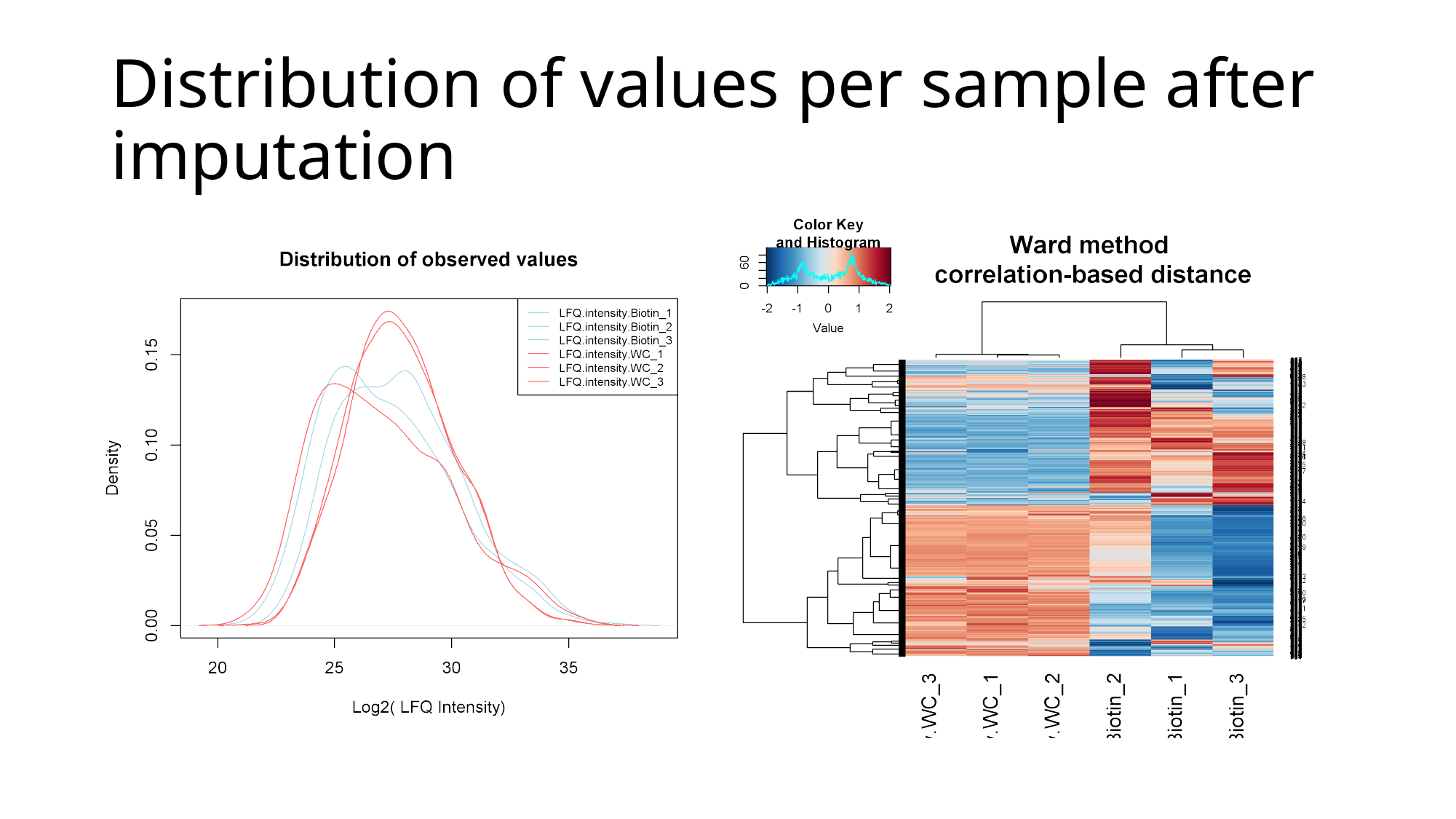

# Distribution of values per sample after imputation

## Slide 11
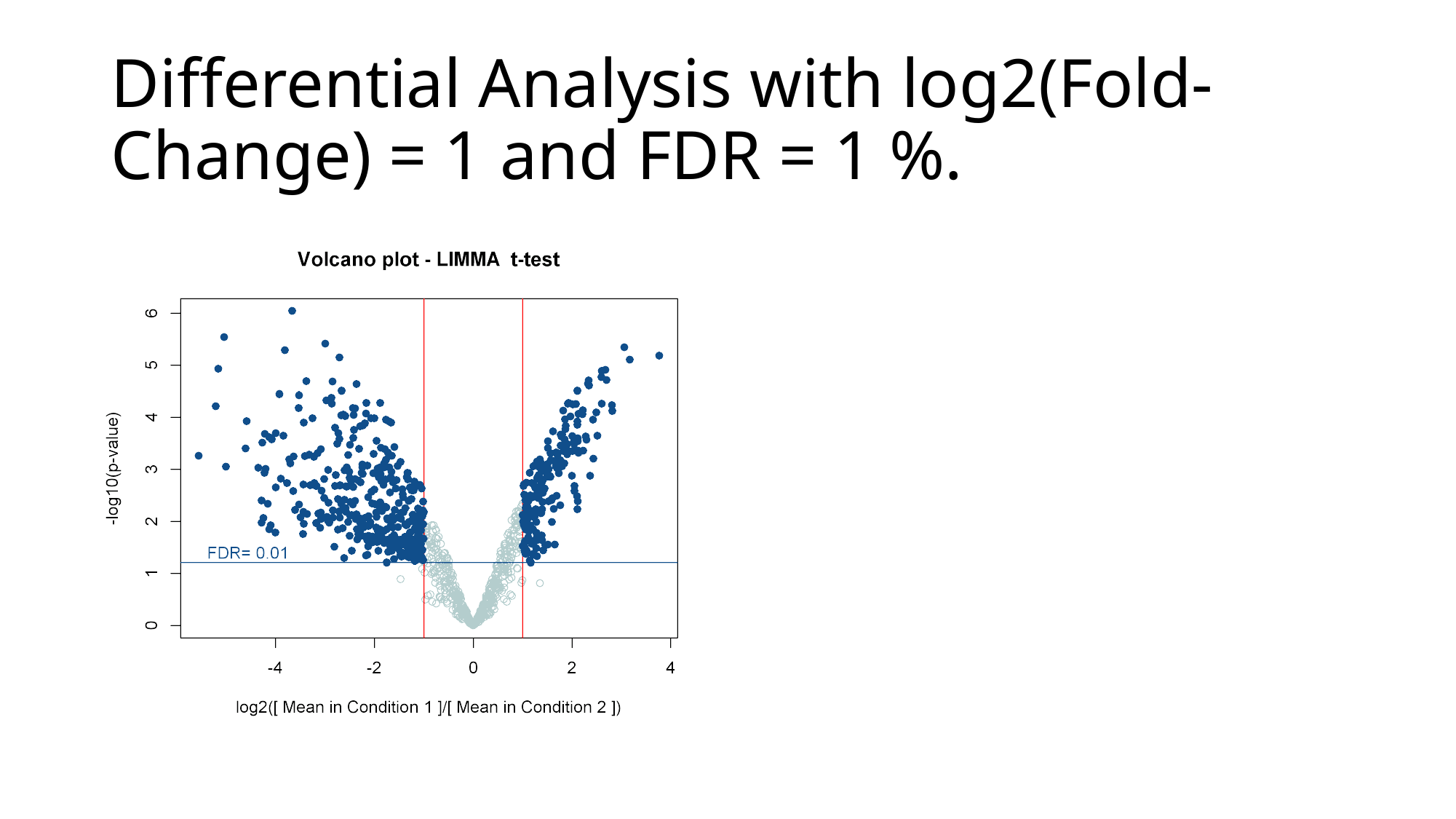

# Differential Analysis with log2(Fold-Change) = 1 and FDR = 1 %.

## Slide 12
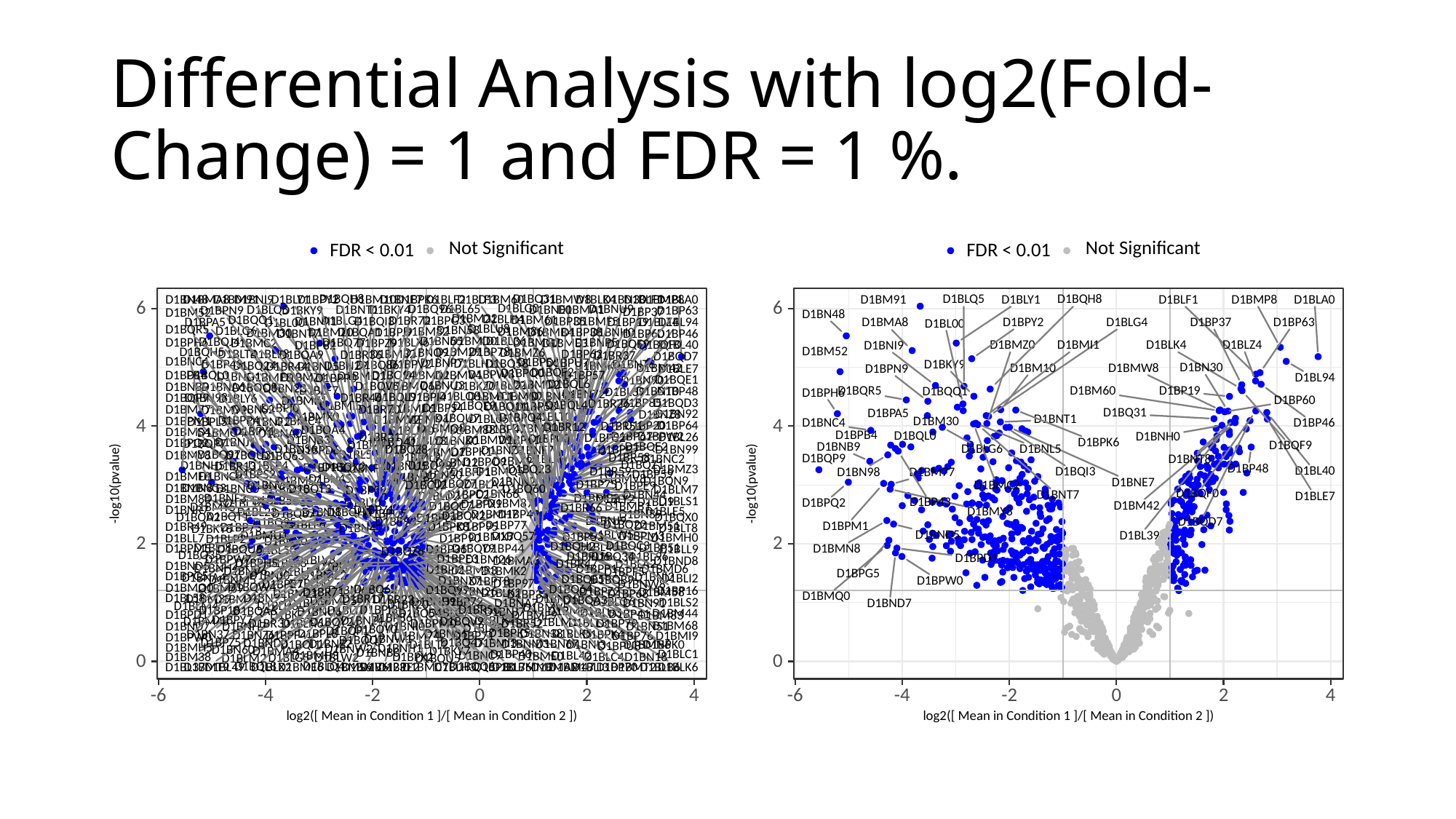

# Differential Analysis with log2(Fold-Change) = 1 and FDR = 1 %.
Not Significant
FDR < 0.01
D1BQH8
D1BQ31
D1BN48
D1BMA8
D1BM91
D1BNI9
D1BM10
D1BPK6
D1BLF3
D1BM60
D1BMW8
D1BN30
D1BMP8
D1BLA0
D1BPY2
D1BLF2
D1BLY1
D1BNE7
D1BLK4
D1BLF1
6
D1BLG0
D1BQ96
D1BNU9
D1BL65
D1BLQ5
D1BNE0
D1BMA1
D1BNT1
D1BKY4
D1BKY9
D1BP63
D1BPN9
D1BP37
D1BM52
D1BM22
D1BLH4
D1BQQ1
D1BM61
D1BR72
D1BQI3
D1BPG3
D1BLG4
D1BMF5
D1BMI1
D1BP35
D1BL94
D1BPA5
D1BLZ4
D1BP19
D1BL00
D1BQR5
D1BLU8
D1BN53
D1BLG6
D1BM82
D1BM86
D1BQA1
D1BPJ9
D1BMZ0
D1BMB4
D1BP38
D1BNH0
D1BM30
D1BNT7
D1BP46
D1BP60
D1BQJ4
D1BL36
D1BN65
D1BMD0
D1BQ77
D1BPH6
D1BMG2
D1BNP5
D1BLX6
D1BPZ9
D1BME3
D1BMC2
D1BQF9
D1BQF0
D1BL40
D1BP82
D1BQH5
D1BNQ9
D1BM29
D1BP78
D1BMZ6
D1BQA9
D1BP62
D1BLF8
D1BM32
D1BLT4
D1BR36
D1BR37
D1BQD7
D1BNC4
D1BP56
D1BQ58
D1BPU7
D1BNP7
D1BLH1
D1BNT8
D1BP43
D1BPV2
D1BQ86
D1BQ24
D1BMK0
D1BN29
D1BNL5
D1BR44
D1BM42
D1BLE7
D1BQF2
D1BPQ0
D1BPW8
D1BQ94
D1BQL0
D1BM12
D1BMV4
D1BP57
D1BPB4
D1BMQ1
D1BNG1
D1BMZ4
D1BME5
D1BPP0
D1BQE1
D1BN90
D1BQL6
D1BMD2
D1BMQ6
D1BNU3
D1BQV5
D1BL22
D1BKZ0
D1BNB9
D1BQQ8
D1BNA6
D1BN23
D1BLF7
D1BP48
D1BNT0
D1BL39
D1BN72
D1BLQ8
D1BN52
D1BMC1
D1BMN7
D1BQL9
D1BQP9
D1BPI4
D1BN98
D1BR46
D1BLY6
D1BMK1
D1BQD3
D1BP85
D1BR26
D1BQL4
D1BQE9
D1BMI5
D1BQ11
D1BP58
D1BPI0
D1BPS4
D1BMB6
D1BM77
D1BNS2
D1BR70
D1BMY9
D1BN92
D1BNZ8
D1BPQ4
D1BLT0
D1BMX0
D1BQU7
D1BL05
D1BM94
D1BMV2
D1BPP4
D1BMC4
D1BPM1
D1BPL5
D1BNE2
4
D1BP64
D1BP20
D1BR51
D1BR12
D1BMQ8
D1BMB0
D1BP47
D1BQA4
D1BM88
D1BLM0
D1BPY1
D1BMS4
D1BMG4
D1BNA0
D1BP67
D1BPW2
D1BR31
D1BL26
D1BP52
D1BP00
D1BP61
D1BMV9
D1BNB3
D1BNK0
D1BLY3
D1BQ41
D1BNJ1
D1BPQ2
D1BQP7
D1BMJ0
D1BQE2
D1BNS6
D1BQZ8
D1BPB7
D1BN99
D1BPD6
D1BNF7
D1BNZ9
D1BPK1
D1BMD7
D1BQ97
D1BL57
D1BQU3
D1BQ63
D1BMY8
D1BML9
D1BR58
D1BLF0
D1BNC2
D1BM93
D1BPQ9
D1BN12
D1BQZ1
D1BQ93
D1BLP4
D1BNH5
D1BR13
D1BNU5
D1BQX3
D1BPP5
D1BNF4
D1BQ23
D1BMZ3
D1BMQ5
D1BR59
D1BPF1
D1BP59
D1BPS2
D1BN80
D1BNC8
D1BMF0
D1BNB2
D1BN45
D1BMV8
D1BQN9
D1BMS7
-log10(pvalue)
D1BNN2
D1BQZ7
D1BLA4
D1BP75
D1BQJ2
D1BNU1
D1BPE9
D1BMN8
D1BNG3
D1BQT3
D1BQ60
D1BNC0
D1BLM7
D1BM70
D1BP02
D1BN66
D1BN44
D1BPC2
D1BLD2
D1BME2
D1BNF3
D1BLY2
D1BM84
D1BLB5
D1BLS1
D1BLI9
D1BLI0
D1BL18
D1BM87
D1BPX9
D1BQC7
D1BMB1
D1BMY5
D1BR66
D1BPZ1
D1BNR7
D1BQH0
D1BLE5
D1BNI8
D1BL23
D1BQ87
D1BPU5
D1BP49
D1BN89
D1BMH7
D1BQX2
D1BQT1
D1BQX0
D1BQA2
D1BPL4
D1BNJ9
D1BP93
D1BQ95
D1BQ22
D1BLF6
D1BP77
D1BM51
D1BP95
D1BR49
D1BPK8
D1BP73
D1BLT8
D1BN41
D1BKY8
D1BLW5
D1BMU1
D1BQ57
D1BPM3
D1BP53
D1BMX7
D1BMH0
D1BP90
D1BLL7
D1BLP2
D1BNX0
2
D1BQC3
D1BQH2
D1BL46
D1BP51
D1BLJ8
D1BQY9
D1BPM5
D1BP44
D1BQU6
D1BP36
D1BLL9
D1BL50
D1BQZ6
D1BQ35
D1BQ34
D1BL96
D1BPU6
D1BPE3
D1BM26
D1BPW7
D1BLV8
D1BMA6
D1BND8
D1BPH5
D1BL08
D1BR47
D1BLA2
D1BND5
D1BLY4
D1BNH4
D1BMD6
D1BPP1
D1BPI2
D1BMB3
D1BMK2
D1BNV6
D1BPF5
D1BLR1
D1BMZ2
D1BNJ0
D1BPG5
D1BNI2
D1BNA4
D1BLI2
D1BQE5
D1BNM5
D1BQB9
D1BNX7
D1BPT8
D1BP97
D1BLH0
D1BNW8
D1BPE7
D1BQW4
D1BMQ0
D1BM69
D1BQ62
D1BQ64
D1BQ99
D1BM62
D1BP16
D1BPZ2
D1BN26
D1BM58
D1BR71
D1BLK2
D1BP68
D1BMZ1
D1BP33
D1BN95
D1BL58
D1BR17
D1BM39
D1BN96
D1BR23
D1BM76
D1BQA3
D1BMD5
D1BLQ6
D1BNH9
D1BP72
D1BLS2
D1BNA7
D1BLJ2
D1BN91
D1BR20
D1BLH6
D1BLM4
D1BMV5
D1BR50
D1BPI3
D1BP18
D1BQA6
D1BLT9
D1BM71
D1BNU6
D1BNJ7
D1BLG7
D1BQ54
D1BLU3
D1BM44
D1BP41
D1BKZ2
D1BPD1
D1BM83
D1BME9
D1BP86
D1BPY7
D1BLX4
D1BQV9
D1BNZ4
D1BM45
D1BQY2
D1BLM1
D1BLL3
D1BR30
D1BR52
D1BPM0
D1BP79
D1BLN6
D1BM68
D1BNI0
D1BN51
D1BND7
D1BNH7
D1BLV9
D1BQY0
D1BQF1
D1BPK5
D1BPL8
D1BN36
D1BN38
D1BLK5
D1BN37
D1BPK4
D1BPF4
D1BNZ6
D1BMI9
D1BP76
D1BM74
D1BP71
D1BPW0
D1BNW1
D1BQQ7
D1BQ47
D1BPZ5
D1BND0
D1BMI3
D1BQI1
D1BN67
D1BNM3
D1BNZ2
D1BNI3
D1BPK0
D1BLT2
D1BMN6
D1BPU8
D1BMH5
D1BNH1
D1BNW2
D1BN60
D1BMA4
D1BKY7
D1BNB0
D1BP40
D1BLC1
D1BL42
D1BNC9
D1BMH3
D1BME0
D1BLC4
D1BN18
D1BM38
D1BPX2
D1BLG5
D1BLW2
D1BQU5
D1BLM2
0
D1BQL8
D1BLQ4
D1BQQ6
D1BL37
D1BMT9
D1BL49
D1BME6
D1BMS6
D1BMV6
D1BM23
D1BLH3
D1BP30
D1BL76
D1BLI3
D1BP70
D1BLB6
D1BLK6
D1BMC7
D1BLX2
D1BPE2
D1BMM2
D1BMA2
D1BMT2
D1BM47
-6
-4
-2
0
2
4
log2([ Mean in Condition 1 ]/[ Mean in Condition 2 ])
Not Significant
FDR < 0.01
D1BLQ5
D1BQH8
D1BM91
D1BMP8
D1BLA0
D1BLY1
D1BLF1
6
D1BN48
D1BP63
D1BP37
D1BPY2
D1BLG4
D1BMA8
D1BL00
D1BMZ0
D1BLZ4
D1BLK4
D1BMI1
D1BNI9
D1BM52
D1BKY9
D1BN30
D1BM10
D1BMW8
D1BPN9
D1BL94
D1BQR5
D1BM60
D1BP19
D1BQQ1
D1BPH6
D1BP60
D1BQ31
D1BPA5
D1BNT1
D1BM30
D1BNC4
D1BP46
4
D1BQL0
D1BPB4
D1BNH0
D1BPK6
D1BQF9
D1BNB9
D1BNL5
D1BLG6
D1BQP9
D1BNT8
D1BP48
D1BQI3
D1BL40
D1BN98
D1BM77
-log10(pvalue)
D1BNE7
D1BMC2
D1BQF0
D1BNT7
D1BLE7
D1BPQ2
D1BP43
D1BM42
D1BMY8
D1BQD7
D1BPM1
D1BND5
D1BL39
2
D1BMN8
D1BPD1
D1BPG5
D1BPW0
D1BMQ0
D1BND7
0
-6
-4
-2
0
2
4
log2([ Mean in Condition 1 ]/[ Mean in Condition 2 ])

## Slide 13
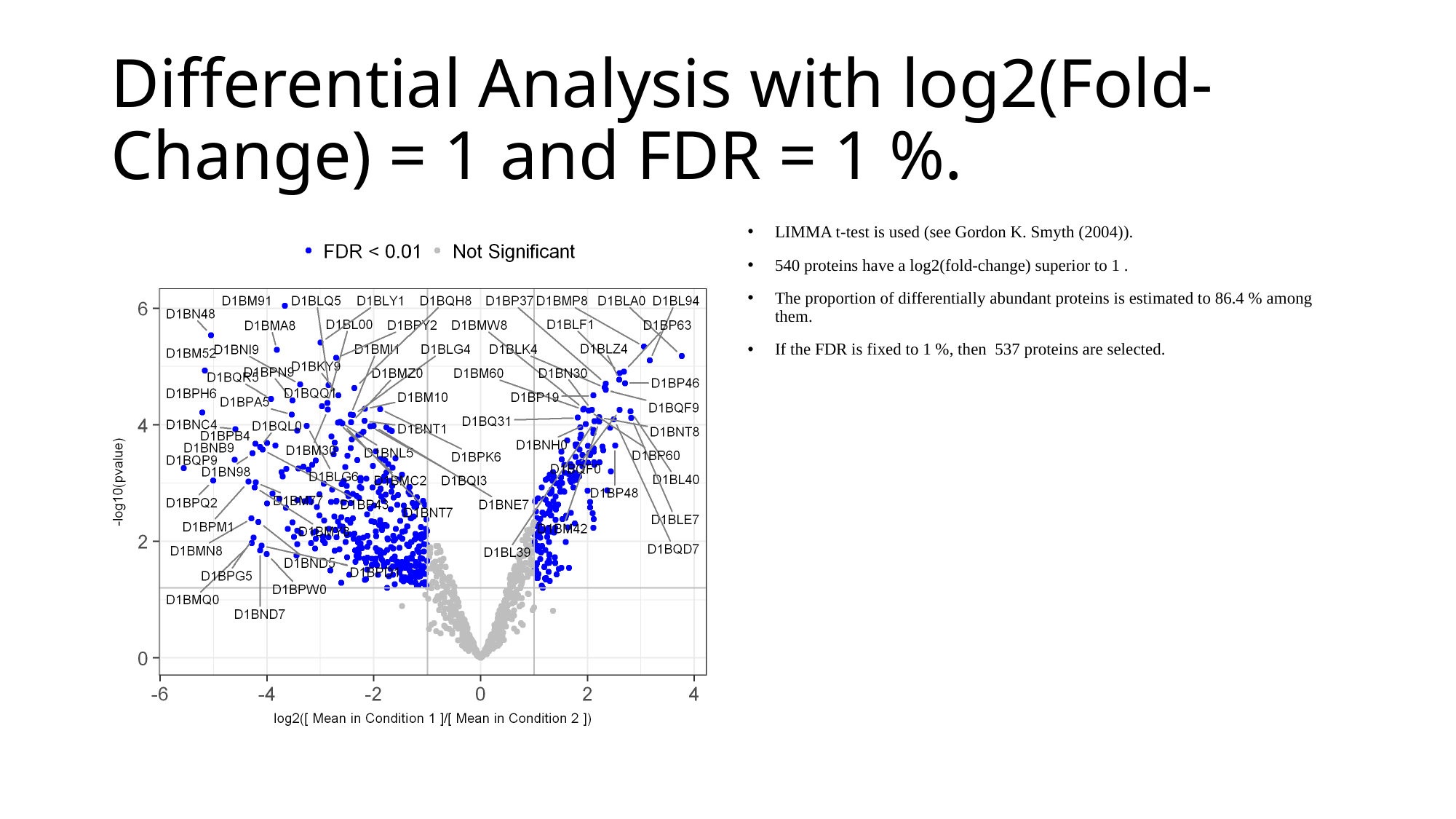

# Differential Analysis with log2(Fold-Change) = 1 and FDR = 1 %.
LIMMA t-test is used (see Gordon K. Smyth (2004)).
540 proteins have a log2(fold-change) superior to 1 .
The proportion of differentially abundant proteins is estimated to 86.4 % among them.
If the FDR is fixed to 1 %, then 537 proteins are selected.

## Slide 14
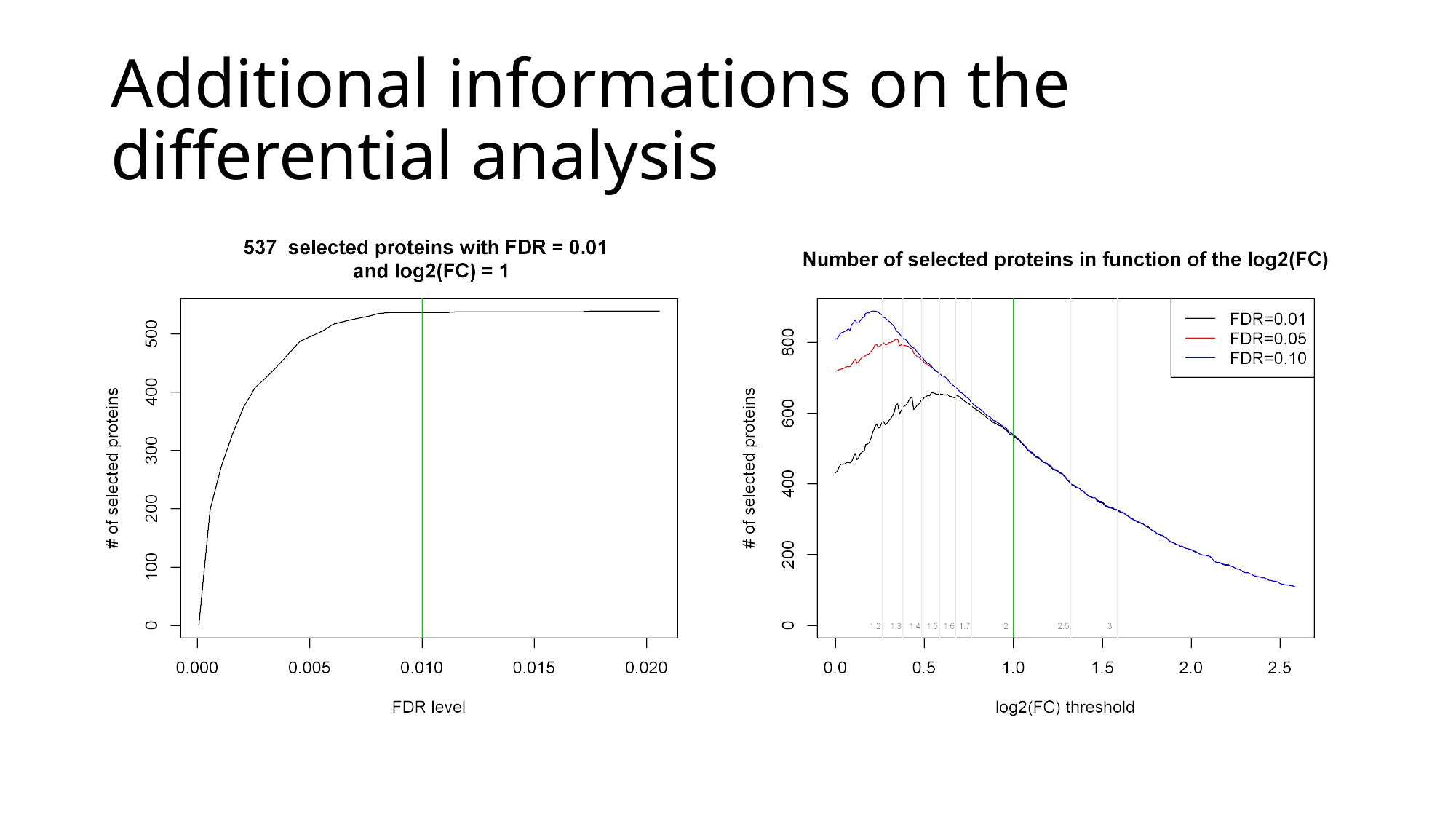

# Additional informations on the differential analysis

## Slide 15
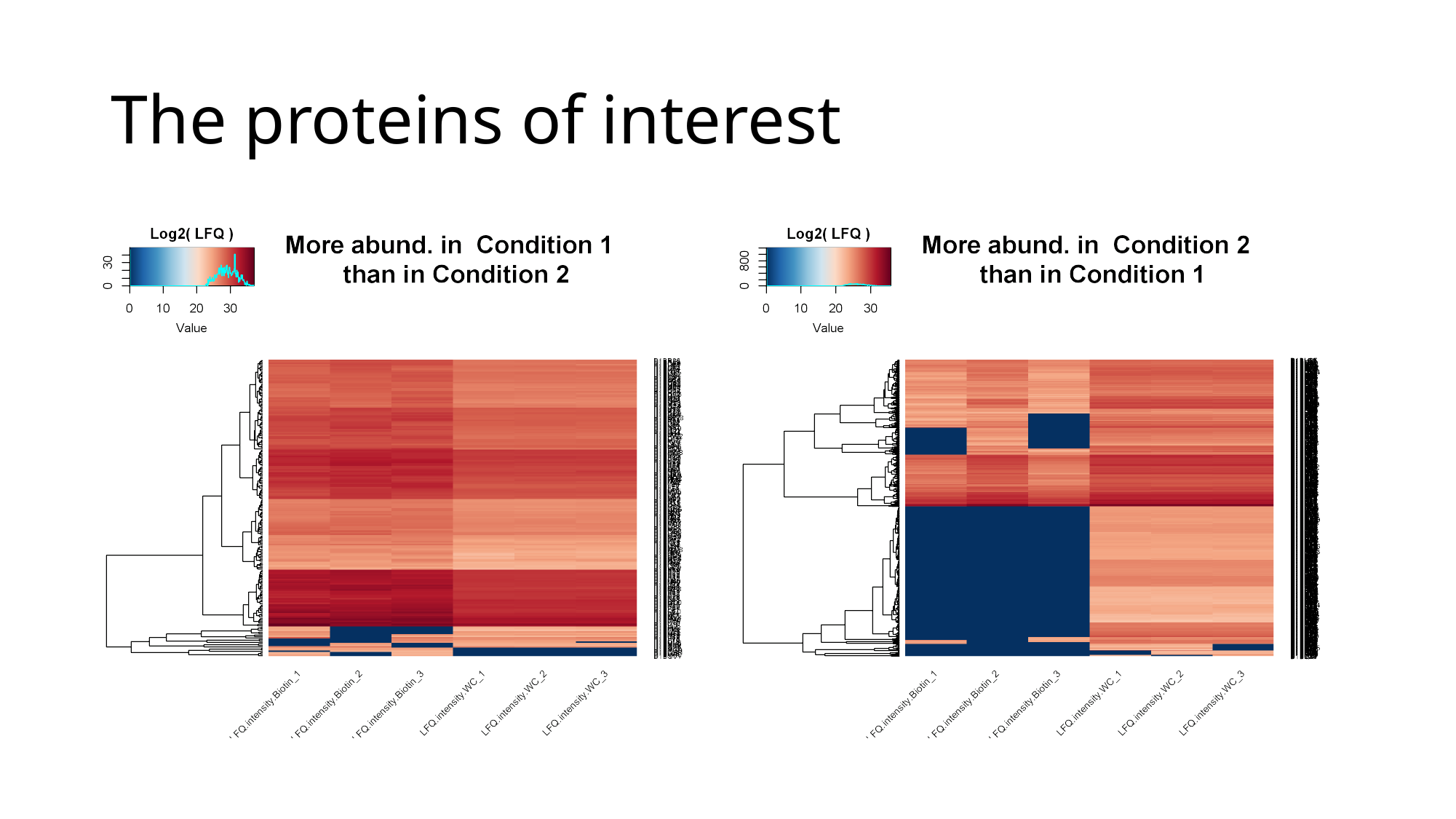

# The proteins of interest

## Slide 16
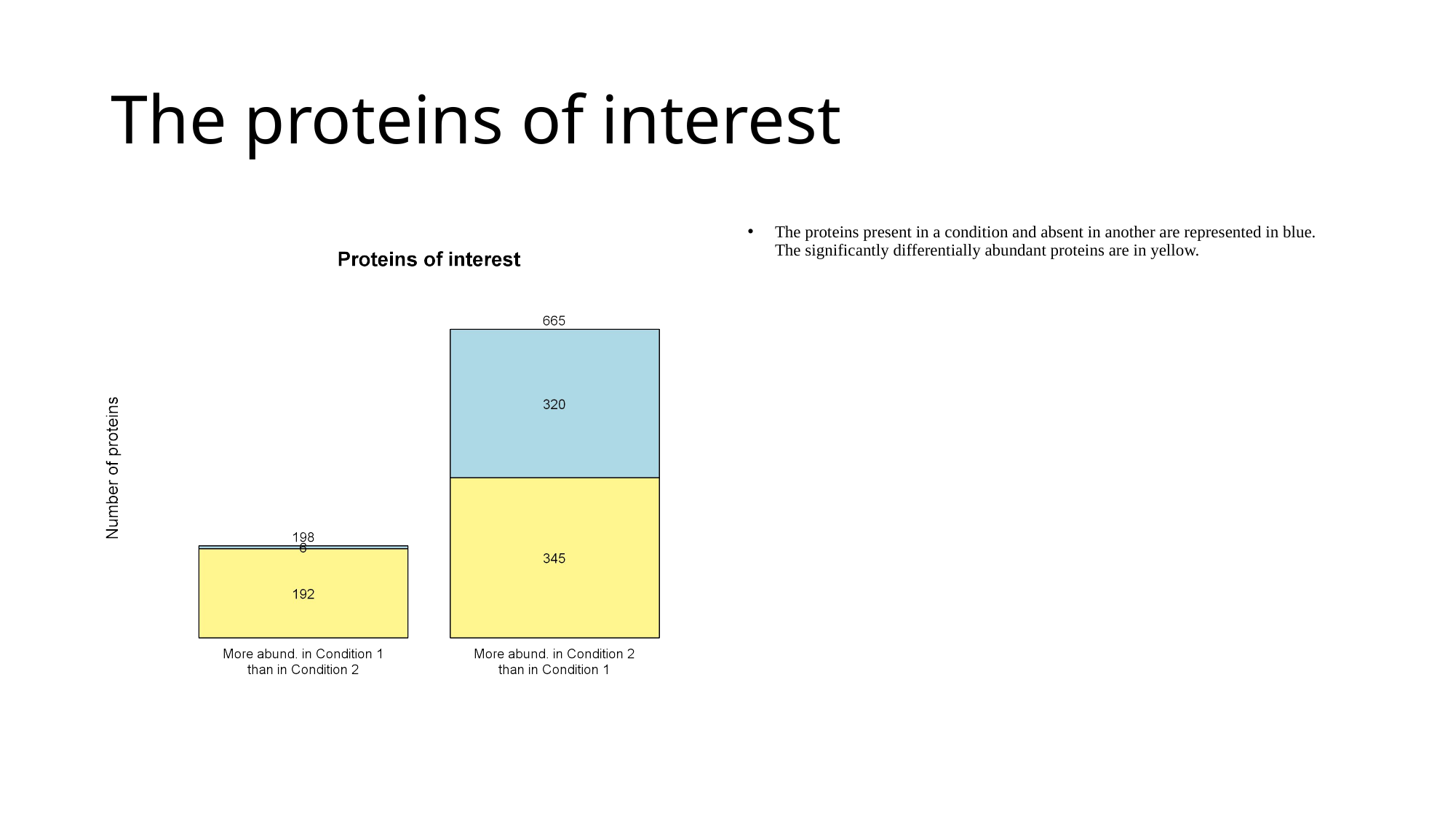

# The proteins of interest
The proteins present in a condition and absent in another are represented in blue. The significantly differentially abundant proteins are in yellow.
